# An HNF1α truncation associated with maturity-onset diabetes of the young impairs pancreatic progenitor differentiation by antagonizing HNF1β function

**DOI:** 10.1016/j.celrep.2022.110425

**Published:** 2022-03-01

**Authors:** Ana-Maria Cujba, Mario E. Alvarez-Fallas, Sergio Pedraza-Arevalo, Anna Laddach, Maggie H. Shepherd, Andrew T. Hattersley, Fiona M. Watt, Rocio Sancho

**Affiliations:** 1 Centre for Stem Cells and Regenerative Medicine, King’s College London, London, UK; 2 Francis Crick Institute, London, UK; 3 University of Exeter Medical School, Exeter, UK; 4 Department of Internal Medicine III, University Hospital Carl Gustav Carus at the Technische Universität Dresden, Dresden, Germany

**Keywords:** MODY3, HNF1α, HNF1β, p291fsinsC, pancreas, diabetes, CRISPR/Cas9, progenitor, organoid, β cell

## Abstract

The HNF1α^p291fsinsC^ truncation is the most common mutation associated with maturity-onset diabetes of the young 3 (MODY3). Although shown to impair HNF1α signaling, the mechanism by which HNF1α^p291fsinsC^ causes MODY3 is not fully understood. Here we use MODY3 patient and CRISPR/Cas9-engineered human induced pluripotent stem cells (hiPSCs) grown as 3D organoids to investigate how HNF1α^p291fsinsC^ affects hiPSC differentiation during pancreatic development. HNF1α^p291fsinsC^ hiPSCs shows reduced pancreatic progenitor and β cell differentiation. Mechanistically, HNF1α^p291fsinsC^ interacts with HNF1β and inhibits its function, and disrupting this interaction partially rescues HNF1β-dependent transcription. HNF1β overexpression in the HNF1α^p291fsinsC^ patient organoid line increases PDX1^+^ progenitors, while HNF1β overexpression in the HNF1α^p291fsinsC^ patient iPSC line partially rescues β cell differentiation. Our study highlights the capability of pancreas progenitor-derived organoids to model disease *in vitro*. Additionally, it uncovers an HNF1β-mediated mechanism linked to HNF1α truncation that affects progenitor differentiation and could explain the clinical heterogeneity observed in MODY3 patients.

## Highlights

• MODY3 patient and CRISPR/Cas9 HNF1α^p291fsinsC^ mutated iPSC lines are generated
• Mutant iPSCs show deficient pancreatic progenitor and β cell differentiation
• Mutant truncated HNF1α protein binds wild-type HNF1β protein to hinder its function
• HNF1β overexpression in MODY3 iPSC line partially rescues β cell differentiation

## Graphical abstract

**Figure:**
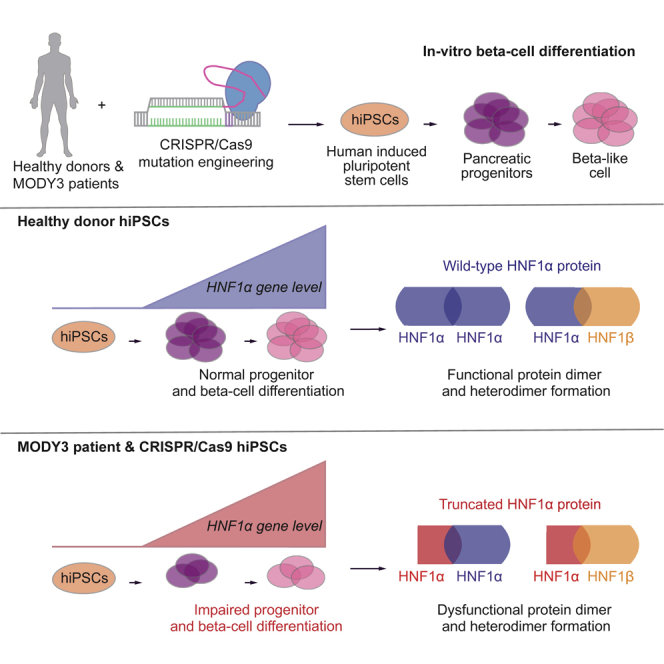

## Teaser

Cujba et al. use human MODY3 patient and CRISPR/Cas9 edited iPSC lines to show that the HNF1α^p291fsinsC^ mutation results in the expression of a mutant truncated HNF1α protein that interacts with wild-type HNF1β protein and inhibits its function, impairing pancreatic progenitor and β cell differentiation.

## Introduction

Maturity-onset diabetes of the young 3 (MODY3) accounts for 30%–70% of all MODY cases. Though classified under the same subtype, more than 200 different heterozygous mutations in the hepatocyte nuclear factor 1α (HNF1α) gene have been associated with MODY3 (Bellanne-Chantelot et al., 2008; Ellard and Colclough, 2006; Frayling et al., 1997; Yamagata et al., 1996). Not surprisingly, patients with MODY3 show a highly heterogeneous clinical phenotype influenced by type and position of HNF1α mutation (Bellanne-Chantelot et al., 2008), patient genetic background (Lango Allen et al., 2010), and environmental effects both before and after birth (Hattersley, 1998; Stride et al., 2002). This heterogeneity is reflected in the treatment required by patients to control diabetes. Patients are initially controlled with diet and sulfonylureas but at variable ages require additional therapy such as insulin (Hattersley, 1998; Lehto et al., 1997; Pearson et al., 2000). However, the molecular mechanism linking specific HNF1α mutations to β cell development remains elusive.

Mutations in the dimerization domain may exhibit different impacts in HNF1α function, related to the impaired binding to co-factors and DNA targets. Mutations in the DNA-binding homeodomain are related to several diseases besides MODY3 (Valkovicova et al., 2019). However, there is not a clear link between reduced DNA binding and reduced transactivation activity, so the impact of these mutations is still uncertain. Mutations in the HNF1α promoter may alter its transcriptional activation, leading to crucial consequences, which are probably the reason for the low rate observed for these mutations. Other mutations that alter the cellular localization of HNF1α and its interaction with other factors and DNA have been found at the transactivation domain (Cubuk and Yalcin Capan, 2021; Valkovicova et al., 2019). Thus, the location of the HNF1α-specific mutation is crucial for the mechanism and exhibited effects.

One of the transactivation domain mutations is HNF1α^p291fsinsC^. This mutation is the most frequent in MODY3 patients and is found in 20%–50% of cases (Frayling et al., 1997; Kaisaki et al., 1997; Yamagata et al., 1996). Insertion of a cytosine (C) in the polycytidine tract of codon 291 (p291fsinsC) of HNF1α results in a frameshift mutation and premature stop codon (Kaisaki et al., 1997). Patients with this mutation exhibit more severe clinical phenotypes than other HNF1α mutated patients, often showing symptoms similar to those of type 1 diabetes (Lebenthal et al., 2018), and in some cases can develop hepatocellular adenoma (Reznik et al., 2004). Therefore, this mutation does not recapitulate other transactivation domain MODY3 mutants. Interestingly, HNF1α^p291fsinsC^ phenotypes in MODY3 have been attributed to a dominant negative effect due to the formation of a truncated HNF1α protein (Okita et al., 1999; Vaxillaire et al., 1999; Yamagata et al., 1998) or haploinsufficiency mediated through nonsense-mediated decay (Harries et al., 2004). However, HNF1α^+/−^ mice do not develop diabetes (Pontoglio et al., 1998) and HNF1α^−/−^ mice show growth retardation, impaired insulin release, and aerobic glucose metabolism, but unlike MODY3 patients suffer from other renal and kidney complications due to the pleiotropic effect of complete loss of HNF1α (Lee et al., 1998; Pontoglio et al., 1996, 1998; Servitja et al., 2009). This shows that mice do not fully recapitulate MODY3 pathogenesis triggered by nonsense and missense mutations. Genetically engineered human embryonic stem cells (hESCs) lacking one or both HNF1α alleles show decreased insulin secretion, glycolysis, mitochondrial function, and developmental bias toward α cells upon differentiation (Cardenas-Diaz et al., 2019). Human induced pluripotent stem cell (hiPSC) lines derived from patients with HNF1α mutations have been generated, but their ability to differentiate into β cells has not been thoroughly addressed (Stepniewski et al., 2015; Yabe et al., 2015, 2019). Interestingly, overexpression of truncated forms of HNF1α in mice results in more severe phenotypes of reduced β cell growth and insulin secretion (Hagenfeldt-Johansson et al., 2001; Yamagata et al., 2002), suggesting that potential truncated forms of HNF1α caused by the p291fsinsC mutation might have additional HNF1α-independent functions, possibly via its interaction with other HNF members.

Even though HNF1α homologous protein HNF1β has a broader tissue expression (Cereghini et al., 1992), the ventral endoderm is the main area affected by homozygous deletion of this protein (Lokmane et al., 2008). Expression of HNF1β appears as early as the primitive gut tube stage of differentiation, and it was demonstrated to be fundamental for the determination of multipotent progenitor cells (De Vas et al., 2015; El-Khairi and Vallier, 2016). Indeed, full deletion of HNF1β expression in mice results in pancreas agenesis, while heterozygous expressing mice have pancreatic hypoplasia and kidney-related phenotypes (De Vas et al., 2015). Similarly, mutations affecting HNF1β in humans cause phenotypes mainly associated with kidney disorders, diabetes mellitus, and MODY (Bockenhauer and Jaureguiberry, 2016).

Although the dimerization between the two homologous proteins has been since long described (Mendel et al., 1991; Rey-Campos et al., 1991) and tissues co-expressing HNF1α and HNF1β display comparable abundance of transcripts (Mendel et al., 1991), implications of an altered interplay between these two proteins are poorly studied (Balamurugan et al., 2016; Kitanaka et al., 2007). Studies on knockout models of either one or the other transcription factor (De Vas et al., 2015; Servitja et al., 2009) alongside general description of affected targets (Verhave et al., 2016) highlighted a set of genes which appear to be regulated by either one or the other, as well as genes potentially regulated by both, therefore hinting at a possible heterodimer dynamic that could affect MODY3 development.

Here, we use an hiPSC-derived progenitor organoid system to understand the mechanism of the p291fsinsC mutation in MODY3. We uncovered a severe developmental defect at the pancreatic progenitor stage in differentiating patient-derived and CRISPR/Cas9-engineered HNF1α^p291fsinsC^ heterozygous hiPSCs, which can be explained by the antagonizing effect of HNF1α^p291fsinsC^ on HNF1β function.

## Results

### MODY3-derived iPSCs show impaired differentiation to pancreas progenitors

**Figure 1:**
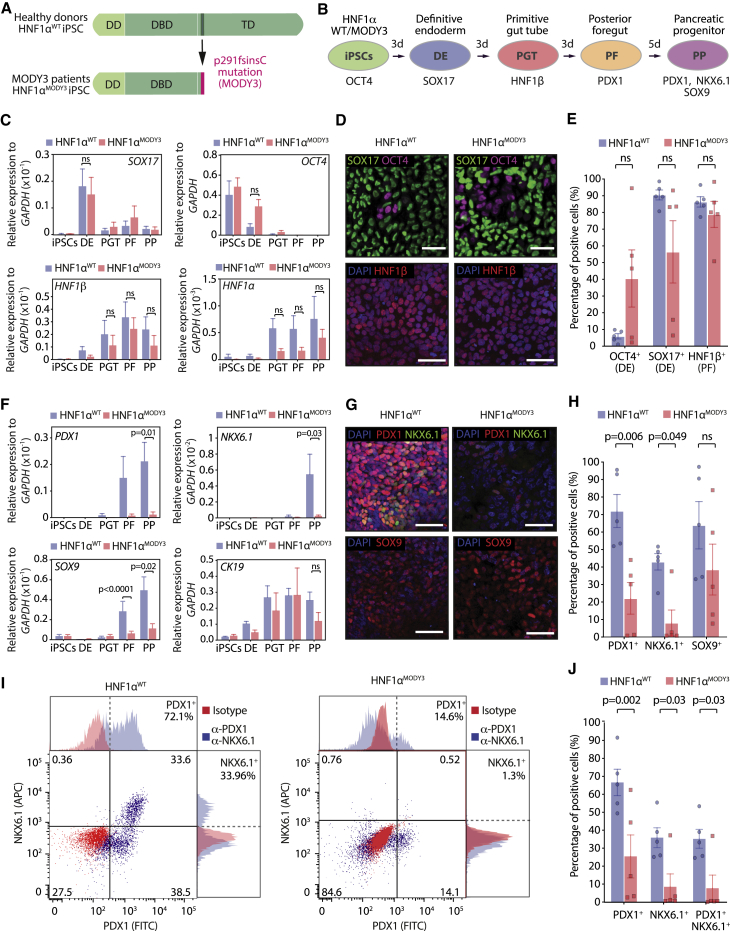
MODY3-derived iPSCs show impaired differentiation to pancreas progenitors (A) Schematic representation of the HNF1α p291fsinsC mutation. (B) Schematic depicting the 14-day differentiation protocol of HNF1α^WT^/HNF1α^MODY3^ iPSCs into PPs. Markers used at each stage are indicated. (C) qRT-PCR analysis of key iPSC, DE, PGT, and PF markers in HNF1α^WT^/HNF1α^MODY3^ lines during differentiation. (D) Representative IF staining for OCT4 and SOX17 (DE stage) and HNF1β (PF stage) in HNF1α^WT^/HNF1α^MODY3^ lines. (E) Quantification of OCT4^+^, SOX17^+^, and HNF1β^+^ cells detected by IF. (F) qRT-PCR analysis of PP markers in HNF1α^WT^/HNF1α^MODY3^ lines. (G) Representative IF staining for PDX1, NKX6.1, and SOX9 at the PP stage in HNF1α^WT^/HNF1α^MODY3^ lines. (H) Quantification of PDX1^+^, NKX6.1^+^, and SOX9^+^ cells detected by IF. (I) Representative flow cytometry dot plot for PDX1 and NKX6.1 co-staining in HNF1α^WT^/HNF1α^MODY3^ PPs. (J) Quantification of PDX1^+^ NKX6.1^+^ PPs from flow cytometry analysis. n = 4–5 individual differentiations (C and F). n = 5 individual differentiations (E, H, and J). Error bars are SEM. p values shown on graphs only for significant differences determined by multiple t tests (C, E, F, H, and J). ns, non-significant. Scale bars, 50 μm (D and G). See also Figures S1–S3.

To investigate whether the HNF1α^p291fsinsC^ mutation in MODY3 patients has any developmental effect on pancreatic progenitor (PP) differentiation, we used five MODY3 patient-derived hiPSC lines (hereafter, HNF1α^MODY3^) alongside five healthy donor hiPSC lines (hereafter, HNF1α^WT^) from the Human Induced Pluripotent Stem Cell Initiative (HipSci) bank (Streeter et al., 2017), confirmed to have no gross chromosomal abnormalities, high pluripotency score, and low novelty score (Figures S1A and S1B). Both sequencing and exome sequencing analysis confirmed that all five studied MODY3 hiPSC lines have the HNF1α^p291fsinsC^ mutation in exon 4 (Figures S1C and S1D). Clinical data confirmed heterogeneous disease phenotypes among the MODY3 patients (Figure S2A). Both HNF1α^WT^ and HNF1α^MODY3^ iPSC lines were differentiated to PPs, showing distinctive cell morphologies at the end of the differentiation (Figure S2B). Upon differentiation toward the pancreatic lineage *in vitro*, both HNF1α^WT^ and HNF1α^MODY3^ lines efficiently reached the definitive endoderm (DE) stage, marked by *SOX17* and decreased *OCT4* expression (Figures 1A–1C). Although residual *OCT4* expression was observed in HNF1α^MODY3^ during DE and primitive gut tube (PGT) stages due to patient-to-patient heterogeneity (Figures 1C–1E and S2C), none was found at later stages of differentiation (Figure 1C). *HNF1α* expression was first detected at the PGT stage and increased throughout posterior foregut (PF) and PP stages in HNF1α^WT^ lines. *HNF1α* expression was slightly, but not significantly, reduced in the HNF1α^MODY3^ lines during PGT and PF, possibly due to nonsense-mediated mRNA decay, as reported previously (Figure 1C) (Harries et al., 2004; Yabe et al., 2019), but recovered at the PP stage to levels comparable with those in the HNF1α^WT^ lines. Despite the slight differences in expression dynamics, both HNF1α^WT^ and HNF1α^MODY3^ lines reached PF stage at comparable levels, as revealed by the similar percentage of HNF1β^+^ cells and *HNF1β* mRNA expression at the PGT and PF stage (Figures 1C–1E and S2D).

While HNF1α^WT^ lines efficiently expressed the PP markers *PDX1*, *NKX6*.*1*, *SOX9*, and *CK19* upon further differentiation to PP stage, HNF1α^MODY3^ lines showed a sharp decrease in *PDX1*, *NKX6*.*1*, and *SOX9* expression (Figure 1F), confirmed by immunofluorescence and flow cytometry analysis of protein levels (Figures 1G–1J and S2E–S2G). Concomitantly, the PDX1/NKX6.1 double-positive progenitor population, crucial for subsequent β cell development (McGaugh and Nostro, 2017; Russ et al., 2015), was reduced 4-fold in HNF1α^MODY3^ lines when compared with HNF1α^WT^ lines (Figures 1J and S2G).

Since some of the HNF1α^MODY3^ lines contained residual OCT4 expression at the DE stage upon differentiation (Figure S2C), we performed enrichment of DE cells for the HNF1α^MODY3^ line that had residual OCT4^+^ cells in the DE stage alongside an HNF1α^WT^ line prior to PP differentiation (Figure S3). At the DE stage, both the HNF1α^MODY3^ and HNF1α^WT^ lines contained a comparable amount of the DE marker CXCR4^+^ cells as detected by immunofluorescence (IF) (Figure S3A). We detached the DE cells and performed magnetic sorting to purify the CXCR4^+^ and CXCR4^−^ fractions (Figures S3B and S3C). We confirmed the enrichment of CXCR4 by qPCR analysis and observed that the HNF1α^MODY3^ line seemed to have higher expression levels of CXCR4 in all the tested fractions when compared with the HNF1α^WT^ line (Figure S3C). We then replated the purified fractions to continue their differentiation to PP. The sorting process clearly affected the ability of both CXCR4^+^ and CXCR4^−^ fractions to reattach. However, while the HNF1α^WT^ line recovered and formed a monolayer after 5 days toward PP differentiation conditions (Figure S3D), the HNF1α^MODY3^ sorted CXCR4^+^ and CXCR4^−^ DE cells failed to reattach and eventually died within 5 days (Figure S3D). Therefore, their potential to differentiate to PP could not be evaluated. However, the levels of OCT4 in the DE stage do not correlate with the PP phenotype severity (measured as percentage of PDX1^+^/NKX6.1^+^ cells) observed in the MODY3 lines (Figure S3E).

Altogether, these data suggest that the heterozygous p291fsinsC mutation in HNF1α^MODY3^ lines impairs PP differentiation, and this is not dependent on the residual *OCT4* expression at DE stage in some of the HNF1α^MODY3^ lines.

### Genetically engineered HNF1α^p291fsinsC^ mutant iPSCs recapitulate the PP differentiation defect observed in MODY3 iPSCs

**Figure 2:**
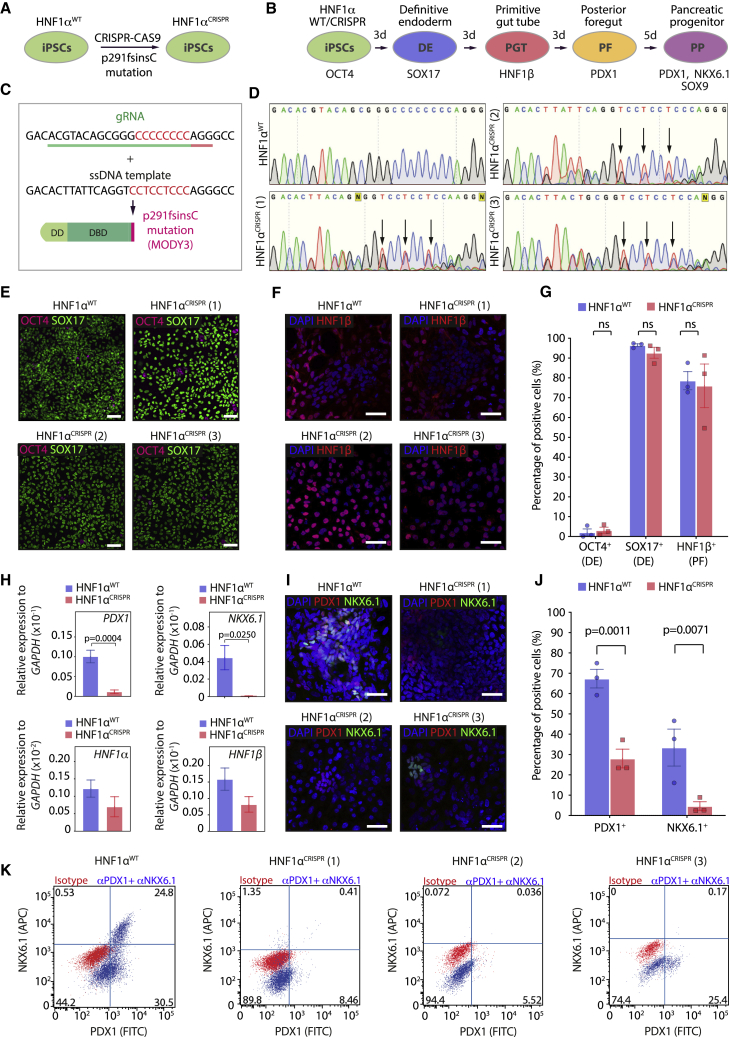
Genetically engineered HNF1α^p291fsinsC^ mutant iPSCs recapitulate the pancreas progenitor differentiation defect observed in MODY3 iPSCs (A) Schematic depicting the strategy to generate HNF1α^CRISPR^ iPSC lines. (B) Schematic depicting the 14-day differentiation protocol of HNF1α^WT^/HNF1α^CRISPR^ iPSCs into PPs. Markers used at each stage are indicated. (C) Guide RNA sequence (green) with protospacer adjacent motif sequence (red) and single-stranded DNA template used to engineer the p291fsinsC mutation in HNF1α^WT^ line. (D) DNA chromatograms of the HNF1α^WT^ iPSC line and the three engineered heterozygous p291fsinsC mutant HNF1α^CRISPR^ iPSC lines. Arrows indicate heterozygosity. (E) Representative IF staining for OCT4 and SOX17 (DE stage) in HNF1α^WT^/HNF1α^MODY3^ lines. (F) Representative IF staining for HNF1β (PF stage) in HNF1α^WT^/HNF1α^CRISPR^ lines. (G) Quantification of OCT4^+^, SOX17^+^, and HNF1β^+^ cells detected by IF. (H) qRT-PCR analysis of *PDX1*, *NKX6*.*1*, *HNF1α*, and *HNF1β* at the PP stage. (I) Representative IF staining for PDX1 and NKX6.1 (PP stage) in HNF1α^WT^/HNF1α^CRISPR^ lines. (J) Quantification of the percentage of PDX1^+^ and NKX6.1^+^ PPs cells detected by IF. (K) Representative flow cytometry dot plot for PDX1 and NKX6.1 co-staining in HNF1α^WT^/HNF1α^CRISPR^ PPs. n = 3 individual differentiations (G, H, and J). Error bars are SEM. p values shown on graphs only for significant differences determined by multiple t tests (G, H, and J). ns, non-significant. Scale bars, 50 μm (E, F, and I). See also Figure S4.

To confirm whether the phenotype observed in the patient HNF1α^MODY3^ lines was solely due to the frameshift mutation in HNF1α and to address the heterogeneity that arises from genetic backgrounds of patient lines, we used CRISPR/Cas9 to engineer the p291fsinsC mutation in an HNF1α^WT^ iPSC line (Figures 2A–2C) and tested its ability to differentiate toward PPs (Figure 2B). Successful genome editing of the HNF1α^WT^ iPSC line was confirmed by sequencing and restriction digest with the newly integrated BseRI restriction site (Figures 2D, S4A, and S4C) in three CRISPR clones harboring the HNF1α p291fsinsC mutation in heterozygosity (HNF1α^CRISPR^). We analyzed the pluripotency of the HNF1α^CRISPR^ lines and their karyotype (Figures S4D and S4E). The three HNF1α^CRISPR^ lines showed OCT4, NANOG, and SOX17 expression, reflecting their pluripotency (Figure S4D). As previously reported for expansion and CRISPR/Cas9 editing of iPSCs *in vitro*, we observed one or two commonly found chromosomal abnormalities (Assou et al., 2020; Rebuzzini et al., 2015) after low-pass sequencing analysis (Figure S4E). However, no abnormalities were detected in chromosome 12, where CRISPR/Cas9 editing of HNF1α locus was performed successfully (Figure S4E). During differentiation into PPs, isogenic HNF1α^WT^ and mutant HNF1α^CRISPR^ lines clones efficiently reached DE and PF stages as marked by an increase in *SOX17* and *HNF1β* expression and downregulation of *OCT4* (Figures 2E–2G). However, similarly to HNF1α^MODY3^ lines, a significant downregulation of *PDX1* and *NKX6*.*1* was observed at the PP stage in all three HNF1α^CRISPR^ clones compared with HNF1α^WT^, with no significant downregulation of *HNF1α* and *HNF1β* observed at this stage (Figure 2H). Furthermore, the percentage of PDX1- and NKX6.1-positive cells in the HNF1α^CRISPR^ clones was reduced by ∼3-fold and ∼10-fold, respectively (Figures 2I and 2J). Accordingly, the percentage of PDX1/NKX6.1 double-positive cells was drastically reduced in all HNF1α^CRISPR^ lines compared with isogenic HNF1α^WT^ lines as detected by flow cytometry (Figure 2K). These data demonstrate that the CRISPR/Cas9-engineered iPSCs fully recapitulate the PP differentiation defect observed in HNF1α^MODY3^ patient iPSC lines.

Additionally, we addressed whether correcting the HNF1α^p291fsinsC^ mutation in the HNF1α^MODY3^ iPSC lines was sufficient to rescue the PP-impaired progenitor phenotype observed. To this end, we performed CRISPR/Cas9 editing in one of the HNF1α^MODY3^ iPSC lines that showed residual OCT4 expression at the DE stage (Figure S4F). We confirmed successful correction of the mutation by sequencing (Figures S4G and S4H). Similar to the CRISPR/Cas9-edited HNF1α^CRISPR^ lines, the HNF1α^CRISPR-CORRECTED^ line showed expression of pluripotency markers NANOG, OCT4, and SOX17 and presented one chromosomal aberration (Figures S4D and S4E). Differentiation of HNF1α^CRISPR-CORRECTED^ line to DE resulted in similar percentage of OCT4^+^ cells when compared with its parental HNF1α^MODY3^ iPSC line (Figures S4I and S4J). However, while the parental HNF1α^MODY3^ line showed a clear impairment in PDX1^+^/NKX6.1^+^ cell differentiation at the PP stage, the HNF1α^CRISPR-CORRECTED^ line showed PDX1^+^/NKX6.1^+^ percentages similar to the HNF1α^WT^ line (Figures S4L and S2G). While correcting the p291fsinsC mutation in the HNF1α^MODY3^ line efficiently reverted the PP defect observed in HNF1α^MODY3^ lines (Figures S4K and S4L), it did not rescue the residual OCT4 levels after differentiation to the DE stage (Figures S4I and S4J). Collectively, our data suggest that the p291fsinsC mutation in HNF1α^MODY3^ induces the PP differentiation defect and that the heterogeneity in DE differentiation among MODY3 lines is patient specific and independent of residual OCT4^+^ cells in DE (Figures 2C–2E and S4I).

### Differentiation of PP-derived organoids to β-like cells is drastically reduced in HNF1α^MODY3^ and HNF1α^CRISPR^ lines

**Figure 3:**
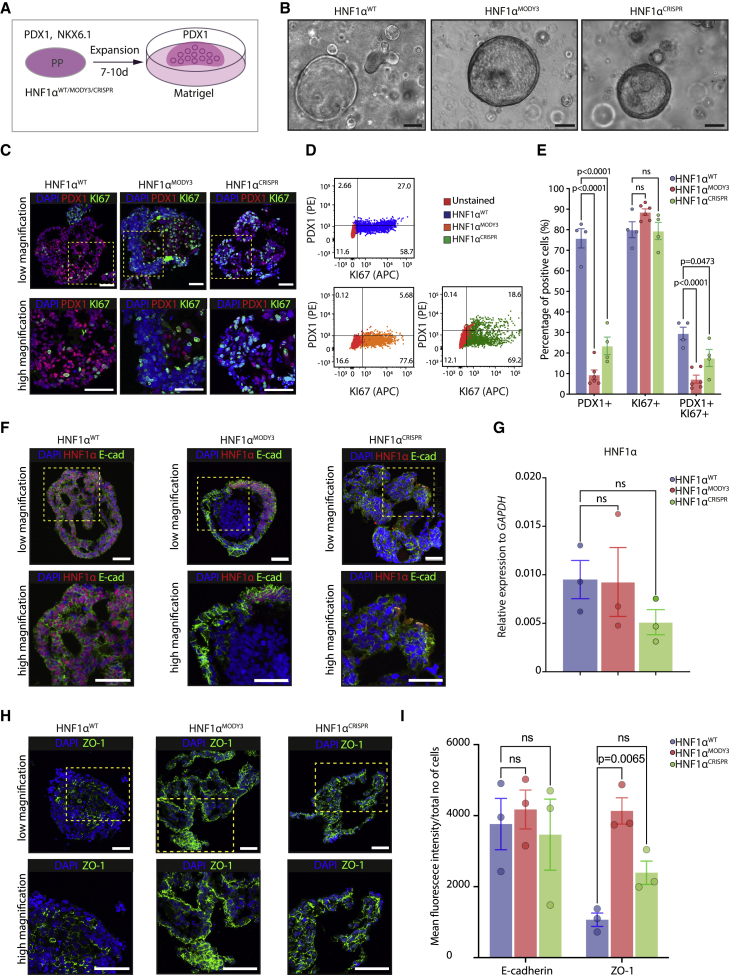
Derivation of pancreas progenitor organoids to study p291fsinsC mutation (A) Schematic depicting the strategy to expand progenitor organoids from HNF1α^WT^/HNF1α^MODY3^/HNF1α^CRISPR^ lines. (B) Representative bright-field images of PP organoids from HNF1α^WT^/HNF1α^MODY3^/HNF1α^CRISPR^ lines. (C) Representative IF staining for PDX1 and KI67 in HNF1α^WT^/HNF1α^MODY3^/HNF1α^CRISPR^ progenitor organoids. (D) Representative flow cytometry staining for PDX1 and KI67 in HNF1α^WT^/HNF1α^MODY3^/HNF1α^CRISPR^ progenitor organoids. (E) Quantification of PDX1^+^, KI67^+^, and PDX1^+^KI67^+^ cells detected by flow cytometry. n = 4–5 independent experiments. (F) Representative IF staining for HNF1α and E-CADHERIN in HNF1α^WT^/HNF1α^MODY3^/HNF1α^CRISPR^ progenitor organoids. (G) qRT-PCR analysis of *HNF1α* in the HNF1α^WT^/HNF1α^MODY3^/HNF1α^CRISPR^ progenitor organoids. n = 3 independent experiments. (H) Representative IF staining for ZO-1 in HNF1α^WT^/HNF1α^MODY3^/HNF1α^CRISPR^ progenitor organoids. (I) Quantification of E-CADHERIN and ZO-1 fluorescence intensity in HNF1α^WT^/HNF1α^MODY3^/HNF1α^CRISPR^ progenitor organoids. Error bars are SEM. p values shown only for significant differences determined by one-way ANOVA followed by Tukey’s multiple comparison test (E, G, and I). ns, non-significant. Scale bars, 100 μm (B) and 50 μm (C, F, and H). See also Figure S5.

One of the key limitations of two-dimensional (2D) models of pancreatic differentiation is the inability to recapitulate the proliferative environment and cell-cell interactions crucial for morphogenesis during pancreas development. We developed a three-dimensional (3D) organoid model system to expand PPs and further characterize the HNF1α^MODY3^ lines. We firstly established HNF1α^WT^ expandable progenitor organoids by adapting published protocols (Boj et al., 2015; Huch et al., 2013). Our method allowed the expansion of PP for at least 15 population doublings (Figures S5A and S5B). Single PP cells formed spheric organoids with a lumen within 7 days in Matrigel (Figure S5C). During the expansion phase, 72.9% of the organoid cells were PDX1^+^ and 8.35% were NXK6.1^+^ (Figure S5D). HNF1α^WT^ organoids retained progenitor marker expression up to passage 15 (Figures S5E and S5F) and efficiently differentiated toward β-like cells in suspension (Figures S5G and S5H). We then established organoid lines from HNF1α^WT^, HNF1α^MODY3^, and HNF1α^CRISPR^ PPs obtained in 2D and analyzed their morphology, marker expression, and proliferative capacity in basal conditions (Figures 3A and 3B). Consistent with results from the PP stage, PDX1^+^ cells were significantly decreased, while levels of proliferation marked by KI67 were maintained at comparable levels. PDX1^+^KI67^+^ cells were significantly decreased in both HNF1α^MODY3^ and HNF1α^CRISPR^ organoids in comparison with HNF1α^WT^ organoids (Figures 3C–3E). Despite comparable gross morphology in all three organoid lines (Figure 3B), HNF1α^MODY3^ organoids showed regions marked by a slight decrease in E-CADHERIN^+^ HNF1α^+^ cells when compared with HNF1α^WT^ organoids (Figure 3F). However, no significant differences in *HNF1*α mRNA levels were observed in HNF1α^MODY3^ or *HNF1*α^CRISPR^ organoids compared with HNF1α^WT^ organoids (Figure 3G). Besides E-cadherin expression, which is known to maintain islet structure and function (Rogers et al., 2007), we also investigated the effect of the p291fsinsC mutation on the tight junction protein Zonula occludens-1 (ZO-1) expression (Figures 3H and 3I). Interestingly, ZO-1 expression had a more homogeneous and higher expression across the HNF1α^MODY3^ and HNF1α^CRISPR^ organoids compared with the apical distribution in the HNF1α^WT^ organoids (Figures 3H and 3I), indicating a potential disorganization of cell polarity in the HNF1α^MODY3^ and HNF1α^CRISPR^ organoids, as has been reported in islets from HNF1α^p291fsins291C^ transgenic mice (Yamagata et al., 2002).

**Figure 4:**
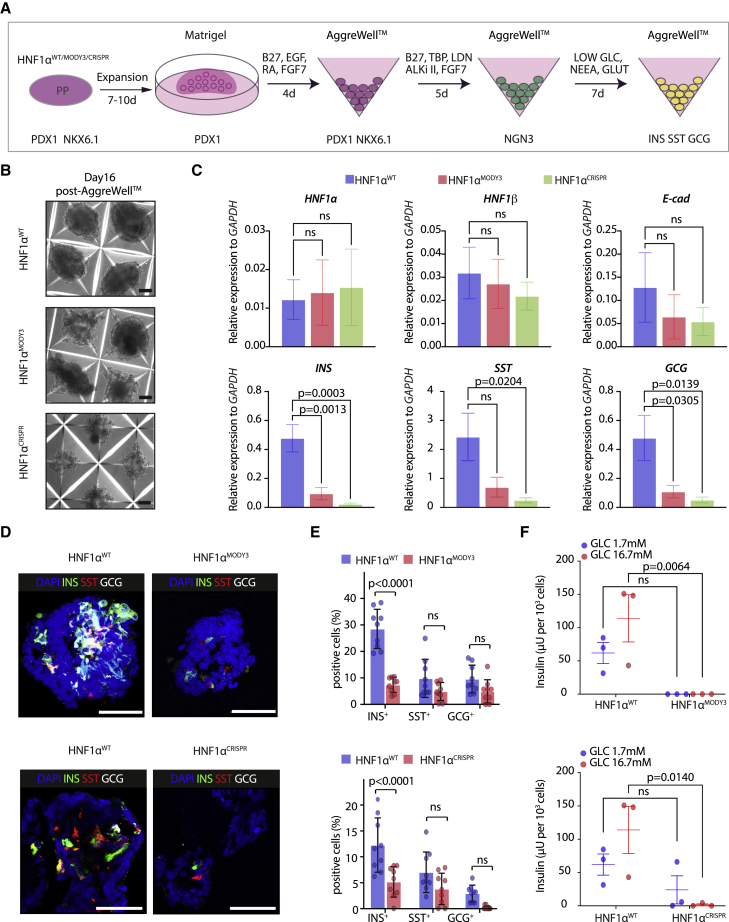
Differentiation of pancreas progenitor-derived organoids to functional β-like cells is reduced in HNF1α^MODY3^ and HNF1α^CRISPR^ lines (A) Schematic depicting the strategy to differentiate PP organoids to β-like cells. (B) Representative bright-field images of HNF1α^WT^/HNF1α^MODY3^/HNF1α^CRISPR^ PP organoids after differentiation. (C) qRT-PCR analysis of *HNF1α*, *HNF1β*, *E-CADHERIN*, insulin (*INS*), glucagon (*GCG*), and somatostatin (*SST*) in differentiated HNF1α^WT^/HNF1α^MODY3^/HNF1α^CRISPR^ PP organoids. n = 5 individual differentiations. (D) Representative IF staining for INS, SST, and GCG in HNF1α^WT^/HNF1α^MODY3^/HNF1α^CRISPR^ organoids. (E) Quantification of INS^+^, SST^+^, and GCG^+^ cells detected by IF. n = 9 organoids from three individual differentiations. (F) Glucose-stimulated insulin secretion (GSIS) comparing HNF1α^WT^ organoid lines versus HNF1α^MODY3^ and HNF1α^CRISPR^ organoid lines, respectively. n = 3 independent differentiations. Error bars are SEM. p values shown only for significant differences determined by multiple t tests (E), one-way ANOVA followed by Tukey’s multiple comparison test (C), or two-way ANOVA followed by Sidak's multiple comparison test (F). ns, non-significant. Scale bars, 100 μm (B) and 50 μm (D).

After differentiation to β-like cells (Figure 4A), HNF1α^MODY3^ and HNF1α^CRISPR^ organoids displayed irregular morphologies with budding cell clusters contrasting the well-defined boundaries observed in HNF1α^WT^ organoids (Figure 4B). In agreement with the reduction of PP markers in HNF1α^MODY3^ and HNF1α^CRISPR^ lines (Figures 1 and 2), a significant decrease in *INS*, *SST*, and *GCG* expression and in the percentage of INS^+^ cells were observed at the endpoint of differentiation of HNF1α^MODY3^ and HNF1α^CRISPR^ organoids to β-like cells when compared with HNF1α^WT^ organoids (Figures 4C–4E). However, no significant differences in *HNF1α*, *HNF1β*, or *E-cadherin* were observed at the mRNA or protein levels between HNF1α^WT^ and HNF1α^MODY3^ lines at the final stage of differentiation (Figures 4C and S5I). Though not absent, INS^+^ cells were reduced from the 30% INS^+^ cells in the HNF1α^WT^ organoids to less than 10% in HNF1α^MODY3^ and HNF1α^CRISPR^ organoids (Figures 4D and 4E). Consistent with the reduction in the percentage of INS^+^ cells in HNF1α^MODY3^ and HNF1α^CRISPR^ organoids, we observed a severe decrease in insulin secretion upon glucose stimulation (Figure 4F), indicating that the resulting INS^+^ cells in the HNF1α^MODY3^ and HNF1α^CRISPR^ organoids are non-functional. Despite of one of the replicas in the HNF1α^CRISPR^ lines showing basal glucose-independent secretion, the secreted insulin levels did not further increase upon high glucose stimulation (Figure 4F). This is consistent with them being non-functional residual β cells. Collectively, these data suggest that the inhibitory effect of the HNF1α^p291fsinsC^ mutation on PP differentiation results in an overall reduction of β-like cell differentiation and function.

### HNF1α^p291fsinsC^ interacts with and inhibits HNF1β-dependent transcription

**Figure 5:**
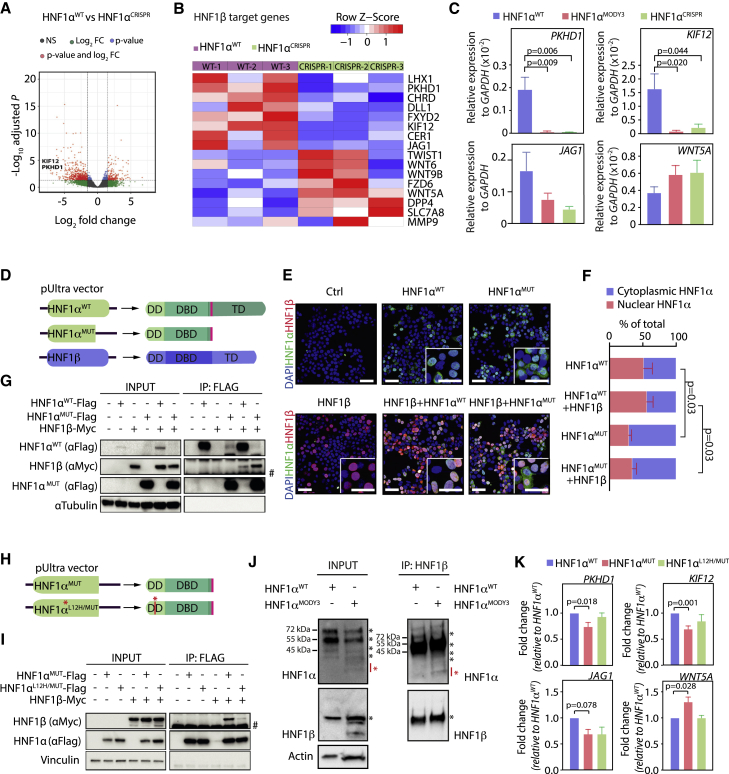
HNF1α p291fsinsC truncated protein interacts with HNF1β and impairs HNF1b-dependent transcription (A) Volcano plot of significantly differentially expressed genes in HNF1α^WT^ versus HNF1α^CRISPR^ PPs. PKHD1 and KIF12 are indicated. (B) Heatmap of differentially expressed HNF1β targets in HNF1α^WT^/HNF1α^CRISPR^ PPs. (C) qRT-PCR analysis of HNF1β target genes *PKHD1*, *KIF12*, *TWIST1*, and *WNT5A*. n = 3 independent differentiations. (D) Schematic depicting the constructs used for overexpression of HNF1α^WT^/HNF1α^MUT^ and HNF1β proteins. (E) IF analysis on HNF1α/HNF1β transfected HEK293T cells. (F) Nuclear/cytoplasmic HNF1α fluorescence intensity quantification in HEK293 transfected with HNF1α^WT^/HNF1α^MUT^ ± HNF1β. (G) FLAG immunoprecipitation in HNF1α/HNF1β transfected HEK293T cells. Vinculin, HNF1β, and HNF1α were detected by immunoblot in the input and immunoprecipitation (IP). n = 3 independent experiments. (H) Schematic depicting the constructs used for HNF1α^MUT^/HNF1α^L12H/MUT^ overexpression. Red asterisk depicts the point mutation L12H known to affect HNF1α dimerization domain. (I) FLAG immunoprecipitation in HNF1α/HNF1β transfected HEK293T cells. Vinculin, HNF1β, and HNF1α were detected by immunoblot in the input and IP. n = 3 independent experiments. (J) HNF1β immunoprecipitation in HNF1α^WT^/HNF1α^MODY3^ PP organoids. HNF1β and HNF1α were detected by immunoblot in the input and IP. Asterisks indicate the specific bands for HNF1β and HNF1α. Red asterisk indicates truncated mutated form of HNF1α. (K) qRT-PCR analysis of HNF1β target genes *PKHD1*, *KIF12*, *JAG1*, and *WNT5A* in HNF1α^WT^ organoids transfected with HNF1α^MUT^/HNF1α^L12H/MUT^ proteins. n = 3–4 independent experiments. Error bars are SEM. p values shown only for significant differences determined by one-way ANOVA followed by Tukey’s multiple comparison test (C, F, and K). Scale bars, 100 μm (E) and 50 μm (E, insets). See also Figures S6 and S7.

To investigate the molecular mechanism explaining the differentiation defects observed at the progenitor stage in HNF1α^MODY3^ and HNF1α^CRISPR^ lines, we performed bulk RNA-sequencing analysis of HNF1α^WT^ and HNF1α^CRISPR^ PPs. Samples within the subgroups clustered closely together by transcriptome analysis (Figure S6A), and HNF1α^CRISPR^ lines showed a decreased enrichment score for β cell gene signature compared with HNF1α^WT^ lines (Figure S6B). Interestingly, among the top 50 differentially expressed genes in HNF1α^CRISPR^ versus HNF1α^WT^ progenitors we found downregulation of *PKHD1* and *KIF12* (Figure S6C), both previously described HNF1β targets, but also reported as affected by HNF1α knockout (Servitja et al., 2009; Chan et al., 2018), supporting a potential homo-/heterodimer dynamic (Figure 5A). HNF1α and HNF1β form homodimers and heterodimers to regulate gene transcription in liver, pancreas, and kidney (Lau et al., 2018) and HNF1β mutations in MODY5 patients leads to severe diabetes (Horikawa, 2018), in part due to the fundamental role of HNF1β in early pancreatic morphogenesis (De Vas et al., 2015; El-Khairi and Vallier, 2016). We hypothesized that HNF1α^p291fsinsC^ could alter the HNF1α-HNF1β balance to affect HNF1α and HNF1β target genes and contribute to the PP differentiation defect observed in HNF1α^MODY3^ and HNF1α^CRISPR^ lines. We found 16 HNF1β target genes described in the literature (Chan et al., 2018; Matsui et al., 2016; Verhave et al., 2016), of which some are involved in Wnt and Notch signaling, and 19 HNF1α-specific targets, as described by Servitja et al. (2009), within the significantly dysregulated genes comparing HNF1α^CRISPR^ and HNF1α^WT^ datasets (Figures 5B, 5C, and S6D). Since the HNF1α^p291fsinsC^ mutation affected both HNF1α and HNF1β target genes, we generated lentiviral-based constructs to overexpress FLAG- or myc-tagged versions of HNF1α^WT^, HNF1α^p291fsinsC^ (HNF1α^MUT^), and HNF1β (Figure 5D) in HEK293T cells and tested for biochemical interaction. While overexpressed HNF1β and HNF1α^WT^ subcellular localization was mainly nuclear, overexpressed HNF1α^MUT^ was present in the cytoplasmic and nuclear fraction and not affected by HNF1β (Figures 5E and 5F). HNF1β efficiently pulled down HNF1α^WT^ and HNF1α^MUT^ in cells co-transfected with HNF1α^WT^/HNF1β or HNF1α^MUT^/HNF1β (Figure 5G).

Since overexpression of HNF1α^MUT^ together with HNF1β or HNF1α^WT^ did not affect HNF1β protein stability measured after a cycloheximide chase (Figures S6E and S6F), HNF1β localization (Figure S6G), or HNF1α^WT^ levels (Figure S6H), we hypothesized that HNF1α^MUT^ could affect HNF1β transcriptional activity by interacting with HNF1β, reducing its ability to homodimerize. To test this hypothesis, we introduced the previously described L12H mutation in the HNF1α^MUT^ dimerization domain (HNF1α^L12H/MUT^) (Narayana et al., 2001) (Figure 5H) and analyzed whether this hampers its interaction with HNF1β (Figure 5I). While HNF1α^MUT^ efficiently interacted with HNF1β, the L12H mutation in HNF1α^L12H/MUT^ was sufficient to abolish its interaction with HNF1β (Figure 5I). Accordingly, HNF1β overexpression in HEK293T cells did not change the subcellular localization of HNF1α^L12H/MUT^ protein, which was mainly observed in the cytoplasm (Figure S7A).

The interaction of HNF1α^p291fsinsC^ and HNF1β proteins could also be detected in patient-derived HNF1α^MODY3^ PP organoids (Figure 5J). Interestingly, overexpressing HNF1α^MUT^ in HNF1α^WT^ PP organoids led to a reduction of the HNF1β target genes PKHD1, KIF12, and JAG1 (Figures 5K, S7B, and S7C). However, overexpressing the HNF1α^L12H/MUT^ dimerization mutant in HNF1α^WT^ PP organoids did not result in a significant difference in the expression of the HNF1β target genes *PKHD1*, *KIF12*, and *JAG1* (Figures 5K, S7B, and S7C), suggesting that the dimerization of HNF1α^p291fsinsC^ and HNF1β is required for the impairment of HNF1β-dependent transcription observed in HNF1α^MODY3^ PPs. To assess the effect of the HNF1α^MUT^ protein on HNF1β binding to the PKHD1 promoter, we performed chromatin immunoprecipitation in cells overexpressing HNF1β, HNF1β^+^ HNF1α^WT^, or HNF1β^+^ HNF1α^MUT^ proteins (Figure S7D), using SATα promoter as a negative control (Figure S7D). We observed that HNF1β efficiently binds to the PKHD1 promoter, and this is enhanced in presence of HNF1α^WT^ (Figure S7D). Interestingly, HNF1β binding to PKHD1 promoter was reduced to baseline levels in the presence of HNF1α^MUT^ (Figure S7D), indicating that the p291fsinsC mutation in the HNF1α^MUT^ protein could impair the binding of both HNF1β-HNF1β homodimers and HNF1β-HNF1α heterodimers to target genes such as PDKH1.

**Figure 6:**
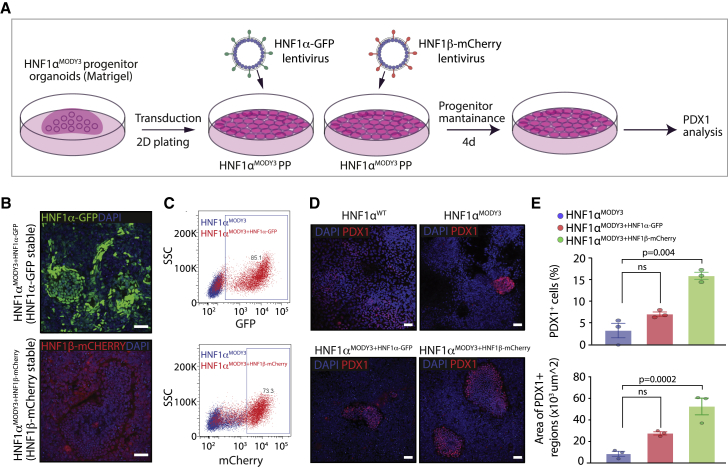
HNF1β overexpression partially rescues the PP defect observed in HNF1α^MODY3^ lines (A) Schematic diagram of the HNF1α-GFP and HNF1β-mCherry lentiviral overexpression strategy in the HNF1α^MODY3^ progenitor organoids. (B) IF analysis of GFP and mCherry in HNF1α^MODY3^ organoids overexpressing HNF1α-GFP and HNF1β-mCherry. (C) Representative flow cytometry dot plot for GFP and mCherry in HNF1α-GFP and HNF1β-mCherry HNF1α^MODY3^ organoids. (D) IF analysis of PDX1 in HNF1α^MODY3^ PP organoids overexpressing HNF1α-GFP and HNF1β-mCherry. (E) Quantification of the percentage and area of PDX1^+^ cells from IF analysis. n = 3 independent experiments. Error bars are SEM. p values shown for significant differences determined by one-way ANOVA followed by Tukey’s multiple comparison test (E). ns, non-significant. Scale bars, 50 μm (B and D).

**Figure 7:**
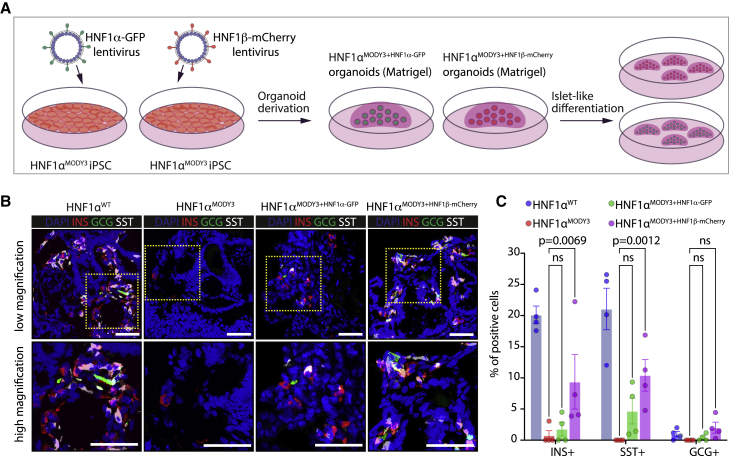
HNF1β overexpression partially rescues the β cell differentiation defect observed in HNF1α^MODY3^ lines (A) Schematic diagram of the HNF1α-GFP and HNF1β-mCherry lentiviral overexpression strategy in the HNF1α^MODY3^ iPSC lines. (B) IF analysis of INS^+^, GCG^+^, and SST^+^ cells on differentiated HNF1α^WT^ organoids, HNF1α^MODY3^ organoids, overexpressing HNF1α^MODY3-HNF1α-GFP^ organoids, and HNF1α^MODY3-HNF1β-mCherry^ organoids. (C) Quantification of the percentages of INS^+^, GCG^+^, and SST^+^ from IF analysis. n = 3 independent experiments. Error bars are SEM. p values shown for significant differences determined by one-way ANOVA followed by Tukey’s multiple comparison test (C). ns, non-significant. Scale bars, 50 μm (B).

Consistent with the effect of the p291fsinsC mutation on both HNF1α- and HNF1β-dependent transactivation and impaired progenitor and β cell phenotype, we further tested whether overexpression of HNF1α or HNF1β using lentivirus transduction would be sufficient to rescue the PP phenotype observed in HNF1α^MODY3^ lines (Figure 6). First, we transduced PP organoids derived from one HNF1α^MODY3^ line with either HNF1α-GFP lentivirus or HNF1β-mCherry lentivirus (Figure 6A) and investigated PDX1 expression after PP differentiation. Efficient overexpression of HNF1α-GFP or HNF1β-mCherry protein was detected by immunofluorescence and flow cytometry analysis in the transduced HNF1α^MODY3^ organoid line (Figures 6B and 6C). Although a partial increase in the percentage of PDX1^+^ progenitors was observed in the HNF1α^MODY3^ line overexpressing HNF1α-GFP or HNF1β-mCherry, only HNF1β-mCherry overexpression led to a significant rescue in the percentage and area of PDX1^+^ cells in the HNF1α^MODY3^ line (Figures 6D and 6E). We further investigated whether HNF1α-GFP or HNF1β-mCherry overexpression was sufficient to rescue the impaired β cell differentiation phenotype observed in HNF1α^MODY3^ lines. To achieve this, we transduced iPSCs with either HNF1α-GFP or HNF1β-mCherry overexpressing lentivirus, generated PP progenitor organoids from the transduced cells, and differentiated them toward islet-like cells (Figure 7A). Consistent with the PDX1^+^ progenitor rescue observed in HNF1β-mCherry overexpressing organoids (Figures 6D and 6E), we detected a significant increase in INS^+^, SST^+^, and GCG^+^ in HNF1β-mCherry overexpressing cells after differentiation when compared with the HNF1α^MODY3^ line (Figures 7B and 7C). Altogether, these data confirm that the interaction of HNF1α^p291fsinsC^ with HNF1β greatly contributes to the progenitor and β cell differentiation defect observed in MODY3 patient iPSC lines.

## Discussion

HNF1α^p291fsinsC^ mutation results in MODY3 diabetes onset with variable penetrance and is mostly diagnosed before 25 years of age (Lebenthal et al., 2018; Urakami, 2019). So far, there has been only limited success in using hiPSCs to understand MODY3 disease mechanisms (Yabe et al., 2019). Interestingly, loss of HNF1α in genetically engineered ESCs shows decreased insulin secretion, glycolysis, mitochondrial function, and developmental bias toward α cells upon differentiation (Cardenas-Diaz et al., 2019). However, whether the complete or partial loss of HNF1α in ESCs recapitulates the most frequent mutation in MODY3 (HNF1α^p291fsinsC^) is not clear.

In this study, by using hiPSCs from healthy donors, MODY3 patients, and CRISPR/Cas9-engineered lines, we have uncovered progenitor and β cell differentiation defects caused by the HNF1α^p291fsinsC^ mutation. Human ESCs lacking both HNF1α alleles develop normally into β cells, with a slight decrease in β cell number and significant downregulation of PDX1 (Cardenas-Diaz et al., 2019). However, our data suggest that the additional HNF1α^p291fsinsC^ dominant negative function over HNF1β accounts for a more severe phenotype not comparable with HNF1α loss, with both HNF1α and HNF1β target genes being affected. Consistently, the HNF1α/HNF1β target genes PKHD1 and KIF12, whose malfunction is associated with developmental defects (Bergmann et al., 2005; Kalantari and Filges, 2020), as well as HNF1β-related components of the Wnt and Notch pathways (Chan et al., 2018) important for early pancreatic development, were downregulated in HNF1α^p291fsinsC^ lines (Figure 5). Our data suggest that by dimerizing with HNF1β, HNF1α^p291fsinsC^ inhibits HNF1β-dependent transcription and PP differentiation.

The severe phenotype observed in the HNF1α^MODY3^ lines is in agreement with previous findings of an earlier onset for patients harboring truncating mutations (Bellanne-Chantelot et al., 2008) and the progressive β cell failure observed in patients over time (Frayling et al., 2001). Though consistently observed, the degree of PP differentiation defect varied among the five patient lines and was associated with the clinical symptoms in these patients, with multiple complications (Figure S2A) and diverse genetic backgrounds contributing to more severe phenotypes (Hattersley, 1998; Lebenthal et al., 2018). Our findings suggest that this heterogeneity could be explained by the antagonizing effect of HNF1α^p291fsinsC^ on HNF1β function (Figure 5). The partial β cell rescue upon overexpression of HNF1β in HNF1α^p291fsinsC^ organoids (Figure 7) implies that HNF1β levels in each patient line could account for the heterogeneity observed and explain distinct MODY3 phenotypes.

Organoid models for progenitor expansion have been developed from mouse and human adult tissue (Huch et al., 2013; Loomans et al., 2018) and hESCs (Bakhti et al., 2019), but have not yet been developed from hiPSCs to model diabetes. Here, we developed an efficient method to expand progenitors from hiPSCs and differentiate them toward β-like cells. By using healthy, MODY3, and CRISPR/Cas9 hiPSC-derived PP organoids, we observed that HNF1α^p291fsinsC^ affects the formation of β-like cells in a relevant human model through a previously undescribed mechanism, with similar phenotypes also observed in mice and pig models of MODY3 (Hagenfeldt-Johansson et al., 2001; Umeyama et al., 2017; Yamagata et al., 2002). Accordingly, patients with HNF1α^p291fsinsC^ mutation display pancreatic abnormalities, with a smaller pancreas size and β cell mass (Sanyoura et al., 2018; Vesterhus et al., 2008), consistent with our results showing a reduced progenitor pool derived from mutant iPSCs known to be a crucial limiting factor for pancreas size and β cell mass (Stanger et al., 2007). In summary, our study identifies a new mechanism by which the truncated HNF1α^p291fsinsC^ protein arising from the common MODY3 p291fsinsC mutation abrogates HNF1β function and impairs PP and β cell development. Additionally, our PP organoid system represents an ideal platform from which to investigate pancreatic developmental defects associated with monogenic diabetes.

### Limitations of the study

Although the number of MODY3 hiPSC lines used in this study was reduced due to the intrinsic difficulty in obtaining hiPSCs from patients with rare diseases, the phenotype was consistent in most patient lines and in the CRISPR/Cas9-engineered lines, allowing us to confidently uncover novel mechanisms of HNF1α^p291fsinsC^ mutation on β cell development. Our *in vitro* model of pancreatic development is reductionist compared with the *in vivo* counterpart tissue, due to absence of exocrine, ductal tissue, and vascularization. In the future, more complex organoid systems that comprise multiple cell types could be used to investigate MODY3 in more physiologically complete models.

## STAR★Methods

### Key resources table

**Table:** 

REAGENT or RESOURCE	SOURCE	IDENTIFIER
**Antibodies**		

Goat anti-SOX17	R&D	Cat#AF1924; RRID: AB_355060
Mouse anti-OCT4	Santa Cruz	Cat#sc-5279; RRID: AB_628051
Rabbit anti-NANOG	CST	Cat#4903S; RRID: AB_10559205
Rabbit anti-HNF1A	CST	Cat#D7Z2Q; RRID: AB_2728751
Goat anti-HNF1B	Invitrogen	Cat#PA5-18642; RRID: AB_10983492
Rabbit anti-HNF1B	Invitrogen	Cat#720259; RRID: AB_2633221
Rabbit anti-PDX1	Cell Signalling	Cat#5679; RRID: AB_10706174
Guinea pig anti-PDX1	Abcam	Cat#ab47308; RRID: AB_777178
Mouse anti-NKX6.1	DSHB	Cat#F55A12; RRID: AB_532379
Mouse anti-NKX6.1	DSHB	Cat#F55A10; RRID: AB_532378
Goat anti-SOX9	R&D	Cat#AF3075; RRID: AB_2194160
Guinea pig anti-insulin	DAKO	Cat#A0564; RRID: AB_10013624
Rabbit anti-somatostatin	DAKO	Cat#A0566; RRID: AB_2688022
Mouse anti-glucagon	abcam	Cat#ab10988; RRID: AB_297642
Mouse Ki67	Cell Signalling	Cat#9449S; RRID: AB_2797703
Mouse CXCR4-A647	BD	Cat#555976; RRID: AB_398616
Rat anti-CK19	DSHB	Cat#TROMA IIIc 10ea; RRID: AB_2133570
Rabbit anti-insulin	abcam	Cat#ab63820; RRID: AB_1925116
Mouse anti-E-cadherin	Cell Signalling	Cat#14472S; RRID: AB_2728770
Rabbit anti ZO-1	Invitrogen	Cat#40-2200; RRID: AB_2533456
Donkey anti-mouse Alexa 647	Invitrogen	Cat#A31571; RRID: AB_162542
Donkey anti-rabbit Alexa 488	Invitrogen	Cat#A21206; RRID: AB_2535792
Donkey anti-goat Alexa 555	Invitrogen	Cat#A21432; RRID: AB_2535853
Donkey anti-rabbit Alexa 555	Invitrogen	Cat#A31572; RRID: AB_162543
Donkey anti-goat Alexa 647	Invitrogen	Cat#A21447; RRID: AB_141844
Donkey anti-mouse Alexa 488	Invitrogen	Cat#A21202; RRID: AB_141607
Goat anti-guinea pig Alexa 488	Invitrogen	Cat#A11073; RRID: AB_2534117
Donkey anti-rat Alexa 647	Invitrogen	Cat#A21472; RRID: AB_1500700
Mouse PDX1-A488	BD	Cat#562274; RRID: AB_10924596
Mouse NKX6.1-A647	BD	Cat#563338; RRID: AB_2738144
Mouse ki67-A647	BD	Cat# 558615; RRID: AB_647130
A488 IgG1k isotype control	BD	Cat#557721; RRID: AB_396830
A647 IgG1k isotype control	BD	Cat#557714; RRID: AB_396823
Anti-Flag	Sigma-Aldrich	Cat#M8823; RRID: AB_2637089
Goat anti-Rabbit HRP	Jackson ImmunoResearch	Cat#111-035-144; RRID: AB_2307391
Donkey anti-Goat HRP	Jackson ImmunoResearch	Cat#705-035-147; RRID: AB_2313587
Goat anti-Mouse HRP	Jackson ImmunoResearch	Cat#115-035-146; RRID: AB_2307392

**Bacterial and virus strains**		

One Shot® TOP10 Competent Cells	Thermo Fisher	C404010

**Chemicals**, **peptides**, **and recombinant proteins**		

Bovine Serum Albumin	Sigma	A1470-100G
Triton X-100	Sigma	T9284-1L
PFA 4%	Alfa-Aesar	J19943
Tween 20	Sigma	P1379
Glycine	Sigma	G7126
Ethylenediaminetetraacetic acid (EDTA)	Fisher BioReagents	BP2482-500
Accutase	BioLegend	423201
Power SYBR Green Master Mix	ThermoFisher	4385614
DAPI (5 mg/ml stock)	Invitrogen	D1306
OCT Embedding Matrix	Fisher	361603E
Cytofix™ Fixation buffer	BD	554655
Pharmingen™ Stain Buffer (FBS)	BD	554656
Phosflow™ Permeabilization Buffer III	BD	558050
Matrigel® Growth Factor Reduced (GFR) Basement Membrane Matrix	Corning	354230
Vitronectin	Stem Cell Technologies	7180
HEPES	ThermoFisher	15630080
Glutamax supplement	ThermoFisher	3505006
Penicilin/Streptomycin	ThermoFisher	15140122
A83-01	TOCRIS	2939
Human FGF-10	Peprotech	100-26
GastrinI	Sigma	SCP0151
Mouse EGF	Peprotech	AF10015
N-acetylcysteine	Sigma-Aldrich	A9165
Nicotinamide	Sigma-Aldrich	N0636-100G
B27 supplement	Thermo Fisher	17504044
Noggin-conditioned-media	This study	N/A
R-spondin-conditioned-media	This study	N/A
Y-27631	Adooq Bioscience	A11001
TryplE	ThermoFisher	12604021
Retinoic acid	Sigma	R2625-50MG
LDN-193189	Stemgent	04-0074
TBP	Millipore	565740-1MG
ALKi II	Axxora	ALX-270-445-M001
NEAA	Gibco	11140035
FGF7	R&D Systems	251-KG-010
Ampicillin	Sigma Aldrich	A9518-25G
Dimethyl sulfoxide (DMSO)	Sigma	D2650-100ML
Proteinase K	VWR	A380,0025
Q5 high-fidelity DNA polymerase	NEB	M0491L
Polyethylenimine (PEI)	Sigma Aldrich	408727
IGEPAL (Nonidet-P40 (NP-40) substitute)	Sigma-Aldrich	I8896
NaF	NEB	P0759
Phenylmethylsulfonyl fluoride (PMSF)	Sigma-Aldrich	PMSF-RO
Orthovanadate	NEB	P0758
Protease inhibitor cocktail	Sigma-Aldrich	P8340-5ML
Laemmli buffer 4x	Genetex	GTX16355
TBS-Tween 20% 20x	Severn	20-7310-10
Polybrene	Merck	TR-1003-G
Lenti-X concentrator	Takara bio	631232
Chelex-100	Biorad	142-1253
PierceTM ChIP grade protein A/G Magnetic beads	Thermo Scientific	26162
PierceTM protein A/G Magnetic beads	Thermo Scientific	88803

**Critical commercial assays**		

RNeasy mini kit	Qiagen	74106
QuantiTect Reverse transcription kit	Qiagen	205314
Spin MiniPrep kit	Qiagen	27106
Spin Midiprep kit	Qiagen	12145
Lipofectamine™ 3000 Transfection Reagent	Thermo Fisher	L3000001
Monarch PCR-cleaning kit	NEB	T1030S
CXCR4 MicroBead Kit	Miltenyi Biotec	130-100-070
MACS LS Columns	Miltenyi Biotec	130-042-401
DirectPCR Lysis Reagent (Cell)	Viagen Biotech	301-C
Bradford assay	Biorad	5000006

**Deposited data**		

Raw and count files have been deposited in NCBI’s Gene Expression Omnibus.	https://www.ncbi.nlm.nih.gov/geo/	GEO: GSE166822
All original code has been deposited at Zenodo and is publicly available as of the date of publication.	https://zenodo.org/	Zenodo: https://doi.org/10.5281/zenodo.5834882

**Experimental models**: **Cell lines**		

Wild-type Human iPSC line	HipSci	HPSI0714i-kute_4
Wild-type Human iPSC line	HipSci	HPSI0514i-toco_5
Wild-type Human iPSC line	HipSci	HPSI0314i-cuhk_1
Wild-type Human iPSC line	HipSci	HPSI0613i-qanu_1
Wild-type Human iPSC line	HipSci	HPSI0314i-hoik_1
MODY3 Human iPSC line	HipSci	HPSI0614i-zoio_1
MODY3 Human iPSC line	HipSci	HPSI0614i-zoio_2
MODY3 Human iPSC line	HipSci	HPSI0614i-guyj_2
MODY3 Human iPSC line	HipSci	HPSI0614i-koqx_1
MODY3 Human iPSC line	HipSci	HPSI0514i-kooz_5
CRISPR Human iPSC line	This study	CRISPR-1
CRISPR Human iPSC line	This study	CRISPR-2
CRISPR Human iPSC line	This study	CRISPR-3
CRISPR Human iPSC line	This study	CRISPR-corrected

**Oligonucleotides**		

Primers for RT-qPCR, see Table S1.	(Cardenas-Diaz et al., 2019); This study	https://www.ncbi.nlm.nih.gov/pmc/articles/PMC6785828/
gRNAs and ssDNA templates for CRISPR-Cas9 strategy, see Table S2.	This study	N/A
Oligonucleotides for constructs used in the biochemical studies, see Table S3.	This study	N/A

**Recombinant DNA**		

pSpCas9(BB)-2A-GFP (PX458)	Addgene (Ran et al., 2013)	plasmid #48138
pUltra backbone	Addgene	plasmid #24129
pMD2.G	Addgene	plasmid #12259
psPAX2	Addgene	plasmid #12260

**Software and algorithms**		

Imaris	Bitplane	https://imaris.oxinst.com/packages
ImageJ	(Schneider et al., 2012)	https://imagej.nih.gov/ij/
RStudio 3.6.1	RStudio	https://www.rstudio.com/
Prism 7 for MacOS	GraphPad	https://www.graphpad.com/scientificsoftware/prism/
FastQC	(Andrews, 2010)	http://www.bioinformatics.babraham.ac.uk/projects/fastqc
CRISPR Finder tool	Wellcome Sanger Institute Genome Editing (WGE) website	https://wge.stemcell.sanger.ac.uk/find_crisprs
Trimmomatic	(Bolger et al., 2014)	http://www.usadellab.org/cms/?page=trimmomatic
STAR	(Dobin and Gingeras, 2015)	https://hbctraining.github.io/Intro-to-rnaseq-hpc-O2/lessons/03_alignment.html
featureCount	(Liao et al., 2013)	https://www.rdocumentation.org/packages/Rsubread/versions/1.22.2/topics/featureCounts
ANNOVAR	(Wang et al., 2010)	https://pubmed.ncbi.nlm.nih.gov/20601685/
Rosalind cluster	King’s College London	https://rosalind.kcl.ac.uk

**Other**		

Essential 8 Basal Medium	Gibco	A15169-01
STEMdiff™ Pancreatic Progenitor Kit	Stem Cell Technologies	05120
Advanced DMEM/F-12	Gibco	12634-010
DMEM with 25 mM Glucose	Gibco	31966-021
DMEM with 2.8 mM Glucose	Gibco	21885-025
Cell recovery solution	Corning	354253
Aggrewell™ 400 plates	Stem Cell Technologies	34411
ProLong® Gold Antifade Mountant	Fisher	P36930
fish gelatin	Sigma	G7765-1L
BbsI restriction enzyme	NEB	R3539S
AgeI restriction enzyme	NEB	R3552S
BseRI restriction enzyme	NEB	R0581S
BamHI restriction enzyme	NEB	R3136S
EcoRI restriction enzyme	NEB	R3101S
T4 DNA ligase	NEB	M0202
PVDF membranes	BioRad	1704156
NaCl	Sigma	S9888
KCl	Riedel de Haën	31248
CaCl_2_	Sigma	C7902
MgSO_4_	Sigma	M2643
Na_2_HPO_4_	Sigma	S0751
KH_2_PO_4_	Sigma	P0662
NaHCO_3_	Sigma	S5761
HEPES (powder)	Sigma	H3375
BSA	Sigma	A9647
Glucose	Sigma	G5767

### Resource availability

#### Lead contact

Further information and requests for resources and reagents should be directed to and will be fulfilled by the lead contact, Rocio Sancho (rocio.sancho@kcl.ac.uk).

#### Materials availability

Resources and reagents will be provided upon request. Materials will be provided upon execution of a suitable materials transfer agreement.

### Experimental model and subject details

Human iPSCs were kindly provided through the Human Induced Pluripotent Stem Cell Initiative (HipSci. For this study, 13 different human iPSC lines were used, five derived from healthy donors: kute4 – female, 25-29 years old; toco5 – female, 55-59 years old; cuhk1 – female, 45-49 years old; qanu1 – female, unknown age; hoik1 – female, 40-44 years old, five derived from MODY3 patients: zoio1, zoio2 – female, 75-79 years old; guyj2 – female, 50-54 years old; koqx1 – female, 25-29 years old; kooz5 – female, 40-44 years old and four generated through CRISPR-Cas9 engineering: CRISPR-1, CRISPR-3 and CRISPR-3, (generated from kute4 – female, 25-29 years old) and CRISPR-corrected, (generated from zoio2 – female, 75-79 years old). Cell authentication and more data on cell lines is available freely through the HipSci website (https://www.hipsci.org/). Human iPSCs were maintained at 37C and expanded in Essential 8 Basal Medium on 6-well plates pre-coated with Vitronectin, with daily media change and passaging every week using 0.5 mM Ethylenediaminetetraacetic acid (EDTA) solution.

### Method details

#### Differentiation of human iPSCs into pancreatic progenitors

Human iPSCs were differentiated into pancreatic progenitors for a period of 14 days using the STEMdiff™ Pancreatic Progenitor Kit following manufacturer’s instructions, with some modifications: cell seeding prior to differentiation was 500,000 cells/well for 12-well plates and 42,000 cells/well for 96-well plates and stage 1 was extended to 3 days.

#### RT-qPCR

Differentiated cells at different stages were detached using Accutase for 10 min at 37C and neutralized with 5% FBS in Dulbecco's phosphate-buffered saline (DPBS). Cells were centrifuged at 300g for 5 min and kept at −80C until RNA extraction. RNA extraction was done using the RNeasy mini kit and cDNA was prepared using QuantiTect Reverse transcription kit according to manufacturer’s instructions with 10ng-13ng of cDNA/reaction with different primers (Table S1). Plate was spun at 1000g for 5 min before running on a CFX 384 Touch RT-qPCR machine. Gene expression was determined by normalization to the housekeeping gene GAPDH. Human islet cDNA was kindly provided by Dr Oladapo Olaniro, Prof Shanta Persaud, Dr Bo Liu, Guo Cai Huang and Pratik Choudhary.

#### Immunocytochemistry of 2D cells

Differentiated cells in 96-well plates at different stages were fixed in 4% paraformaldehyde solution diluted in D-PBS for 20 min at 4C, followed by wash and storage in D-PBS. Alternatively, 2D progenitors and HEK293A cells were cultured on 12-well glass bottomed plates and fixed as above. Cells were washed three times in D-PBS and incubated in 100 μl of 0.1-0.5% Triton X-100 in D-PBS with 10% donkey serum for 20 min at RT. Cells were incubated with 100 μl of 0.1-0.5% Triton X-100 in D-PBS with 1% donkey serum containing primary antibodies overnight at 4C. Cells were washed three times in D-PBS and then incubated with 100 μl of 0.1-0.5% Triton X-100 in D-PBS with 1% donkey serum containing secondary antibodies for 1 hr at RT. Cells were washed three times in D-PBS and DAPI (1:1000) was added to the first wash. Stained cells were visualized using the Operetta CLS™ High-Content Analysis System or the Leica SP8 confocal microscope.

#### Immunohistochemistry of 3D organoids

Expanding organoids in Matrigel were washed twice in ice-cold D-PBS and retrieved from Matrigel using 500 μl Cell recovery solution through incubation for 30 min on ice, followed by neutralization with cold DMEM, D-PBS and spinning at 4000g for 5 min. From this stage, both retrieved expanded organoids or differentiated organoids in Aggrewell plates were fixed in 1 ml of 4% paraformaldehyde solution in D-PBS for 30 min on ice. The organoids were washed thrice in cold D-PBS and either maintained at 4C or embedded for sectioning. For embedding, the organoids in D-PBS were equilibrated in an equal volume of 30% sucrose for 15 min at RT. OCT Embedding Matrix was added up to the 750 μl mark without bubbles and sucrose was diluted by using a pipette tip to swirl gently. Tubes were spun in both directions at 3000g for 4 min in a microcentrifuge, allowing organoids to settle at the bottom of the tubes. Organoids were aspirated using a P1000 cut pipette tip and transferred to a prepared OCT-containing chalk in a recognizable column. Chalks were transferred to dry ice to allow solidification and further kept at -80C or used for cryosectioning into 10 μm sections. For staining, slides were incubated with 0.5% Triton X-100 in D-PBS for 5 min at RT in dark. Sections were then incubated for 1hr at RT in the dark with blocking buffer containing PBS, 10% fetal bovine serum (FBS), 3% bovine serum albumin (BSA), 0.05% Triton X-100, 0.05% Tween 20 and 0.25% fish gelatin. Primary antibodies were diluted accordingly in blocking buffer and added to the sections overnight at 4C in the dark. Next day, sections were washed thrice with D-PBS and incubated with secondary antibodies and DAPI (1/1000) for 1-2h at RT in the dark. Sections were washed thrice with D-PBS and mounted using ProLong® Gold Antifade Mountant and a coverslip.

#### Flow cytometry analysis

At the end of pancreatic progenitor stage, differentiated cells were detached using Accutase for 10 min at 37C, neutralized with 5% FBS in DPBS, centrifuged at 300g for 5 min and fixed using the Cytofix™ Fixation buffer for 20 min at 4C. Cells were washed in Pharmingen™ Stain Buffer (FBS) and at least 100,000 cells were used per staining. Cells were permeabilized using the Phosflow™ Permeabilization Buffer III on ice for 30 min. Cells were washed twice in Stain Buffer and incubated with 100 μl Stain Buffer containing conjugated antibodies for 30 min at RT in the dark. Cells were washed three times with Stain Buffer, filtered and analysed using Canto1 machine.

#### Expansion of pancreatic progenitor organoids

At the end of differentiation into pancreatic progenitors, cells were detached using Accutase for 10 min at 37C, neutralized with 5% FBS in DPBS and centrifuged at 300g for 5 min. The cell pellet was resuspended in approximately 300 μl Growth Factor Reduced (GFR) Matrigel® and 25 μl of the cell culture suspension was seeded in pre-warmed 24-well plates. Plates were incubated for 10 min at 37C to allow solidification. 500 μl of Expansion Media containing Advanced DMEM/F-12, 1M HEPES, 1XGlutamax supplement, 1% Penicillin/Streptomycin, 0.5μM A83-01, 0.05 μg/ml mouse EGF, 0.1 μg/ml human FGF-10, 0.01 μl Gastrin, 1.25mM N-acetylcysteine, 10mM Nicotinamide, 1XB27 supplement and 10% Noggin and R-spondin-conditioned-media with 10.5μM Y-27631 as published by others (Boj et al., 2015; Huch et al., 2013) was added to each well for the first 2-3 days. Media was changed every 2-3 days excluding Y-27631. Organoids were expanded for 7-10 days before splitting. Organoids were split using TryplE for 5 min on a 37C shaker. D-PBS was added to neutralize TryplE, cells were centrifuged at 5000g and resuspended in Matrigel (1/4 or 1/6 split ratio) and expanded as described above. Organoids were expanded up to passage 15, frozen at different passages in Expansion Media with 10% dimethyl sulfoxide (DMSO) and went through freeze-thaw cycles with good recovery.

#### Differentiation of pancreatic progenitor organoids

Organoids were split into single cells using TryplE and seeded at 375,000 cells/well in Aggrewell™ 400 plates in Expansion Media with 10.5μM Y-27631. Next day, cells were differentiated for 16 days adapting the protocol from Russ and colleagues (Russ et al., 2015) with modifications implemented by Trott and colleagues (Trott et al., 2017): 2 days - DMEM with 25 mM Glucose with 1xB27, 50 ng/ml EGF, 1uM RA; 2 days - DMEM with 25 mM Glucose with 1xB27, 50 ng/ml EGF, 50 ng/ml FGF7, 5 days - DMEM with 25 mM Glucose with 1xB27, 500 nM LDN-193189, 30 nM TBP, 1000 nM ALKi II, 25 ng/ml KGF; 7 days - DMEM with 2.8 mM Glucose, 1:100 Glutamax, 1:100 NEAA. Media change was done daily with 1 ml of media with double amount of growth factors, while cells were maintained on orbital shaker at 100 rpm. Alternatively, domes of Matrigel were detached and transferred to a 6-well plate with 3 ml of differentiation media, following the same protocol as describe above.

#### CXCR4 sorting at definitive endoderm stage

Differentiated iPSCs at DE stage were sorted using CXCR4 MicroBead Kit (Miltenyi Biotec) following manufacturer’s instructions. Briefly, DE cells were harvested using TryplE, washed with PBS supplemented with BSA and DNAse, and counted. Then, cells were immunostained with CXCR4-APC antibody for 10 min at 4C. Immunostained cells were washed and mixed with Anti-APC MicroBeads for 15 min at 4C. After washing, cells/beads mix was passed through MACS LS Columns (Miltenyi Biotec) to perform magnetic separation. CXCR4 positive and negative cells were directly re-seeded in vitronectin coated plates to continue their differentiation to PP.

#### Glucose stimulated insulin secretion (GSIS)

GSIS was performed to study the ability of differentiated cells to release insulin in response to glucose, as previously described (Truchan et al., 2015). Briefly, at the end of the differentiation, the clusters were washed with PBS and equilibrated for 1h in Krebs buffer (NaCl 129mM, KCl 4.7mM, CaCl2 2.5mM, MgSO4 1.2mM, Na2HPO4 1mM, KH2PO4 1.2mM, NaHCO3 5mM, HEPES 10mM, and BSA 0.1% in distilled water) with 1.7mM glucose. Then, cells were incubated with Krebs buffer with 2.8mM and 16.7mM glucose, 1h each. Next, clusters were dispersed with TryplE for 5 min and cells were counted using a haemocytometer. The insulin in the supernatants was quantified using Ultrasensitive Insulin ELISA kit (Mercodia).

#### CRISPR/Cas9 gRNA and ssDNA design

The guide RNA (gRNA) was designed to cut the wild-type sequence adjacent to the polyC tract next to a protospacer adjacent motif (PAM) sequence using the CRISPR Finder tool from the Wellcome Sanger Institute Genome Editing (WGE) website (https://wge.stemcell.sanger.ac.uk/find_crisprs) on chromosome 12 at exon 4 of HNF1α gene. The gRNA pairs were designed with sticky BbsI ends for downstream integration into the pSpCas9(BB)-2A-GFP (PX458) plasmid (Addgene, plasmid #48138) (Ran et al., 2013), kindly provided by Dr Victor Augusti Negri. To induce the p291fsinsC mutation, single-strand (ss)DNA template were designed with the PAM silently mutated to avoid destruction by the Cas9 enzyme and harbouring a new BseRI restriction site to allow downstream screening.

#### CRISPR/Cas9 gRNA cloning into PX458 plasmid

The gRNA pairs with sticky BbsI ends were annealed in a thermocycler (95C for 5 min and then ramped down to 25C by 5 C/min). The pSpCas9(BB)-2A-GFP (PX458) plasmid was digested with BbsI restriction enzyme, and the ligation components were added to the digested plasmid using T4 DNA ligase according to manufacturer’s instructions. The ligation reaction was transformed in One Shot® TOP10 competent cells according to manufacturer’s instructions. Bacteria were plated in Luria-Bertani (LB) agar plates containing 100 μg/mL of ampicillin and incubated at 37C for 12-14h. Colonies were picked and expanded overnight in 5 ml LB broth at 37C. Next day, the LB solution was used for plasmid purification with the MiniPrep kit following manufacturer's instructions. DNA content of the purified plasmids was quantified using the NanoDrop 2000 spectrophotometer. For checking the integration of gRNAs into the PX458 plasmid, restriction digest reactions were set up with the BbsI and AgeI enzymes as per manufacturer’s instructions.

#### Transfection of iPSCs with gRNA-PX458 plasmid construct and ssDNA templates

Human iPSC lines were expanded up to 70-90% confluency in 6-well plates prior to transfections using Lipofectamine 3000 according to manufacturer’s instructions. 3μg of gRNA-PX458 plasmid and 200pM ssDNA templates were simultaneously transfected in a wild-type line in presence of E8 with 10.5μM Y-27631 to generate mutant iPSC lines. Cells were checked after 24h under a fluorescent microscope, media was replenished, and cells were allowed to recover for three days before sorting.

#### FACS-sorting and screening of transfected iPSCs

Transfected iPSCs were prepared for fluorescence-activated cell sorting (FACS) sorting by pre-incubation with fresh E8 media with 10μM Y-27631 for at least two hours. Cells were dissociated using Accutase for 10 min at 37C and the cell suspension was neutralized using 10% FBS in D-PBS. Cells were centrifuged for 3 min at 200g and resuspended in FACS-sorting buffer (1x Hank's Balanced Salt Solution (HBSS), 1mM EDTA, 25mM 2-[4-(2-Hydroxyethyl)-1-piperazinyl]-ethanesulfonic acid (HEPES), 3% FBS and 10μM Y-27631). Live cells were sorted under sterile conditions using the BD Aria 3 in 96-well plates or as serial dilutions in a 6-well plate. Plates were centrifuged for 3 min at 70g to ensure attachment and incubated at 37C for 48h in E8 media with 10μM Y-27631. Media was changed daily until colonies of 30-50 cells appeared, from when the media was changed to plain E8 media. Individual colonies were picked and transferred to 24-well plates and subsequently to 12-well plates for expansion. Upon confluency, 70% of the well was frozen in E8 with 10% DMSO and 30% of the well was centrifuged for 3 min at 200g and used for screening using Sanger sequencing or/and PCR-based enzyme digestion. Genomic DNA was extracted using DirectPCR Lysis Reagent (Cell) containing 1 mg/ml Proteinase K. Eppendorf tubes were incubated at 55C for 6h (or overnight) at 500 rpm on a Thermomixer, followed by incubation at 85C for 45 min and on ice for 5 min. Eppendorf tubes were centrifuged at 13,000 rpm for 5 min and the supernatant was transferred to a new Eppendorf tube, which was further used for PCR/Sanger sequencing (0.5 μl/PCR reaction of 25 μl). Flanking primers were designed to generate a 277 bp fragment around the polyC tract. The fragment was amplified using Q5 high-fidelity DNA polymerase kit according to manufacturer’s instructions on a PCR machine. The generated PCR fragments were either ran by DNA electrophoresis or cleaned using the Monarch PCR-cleaning kit and further sequenced by Sanger sequencing using a sequencing primer spanning the generated PCR fragment. Positive clones were expanded and differentiated towards pancreatic progenitors and beta-like cells.

#### Cloning

Expression of HNF1α/HNF1α^MUT^ proteins was achieved by cloning the wild-type HNF1α coding sequence (CDS) and the truncated HNF1α CDS into a pUltra plasmid, with a Flag tag added to allow identification and immunoprecipitation. HNF1β CDS was cloned into a mCherry-pUltra plasmid, where a myc tag was added. For both proteins BamHI and EcoRI were used as restriction sites. Point mutagenesis into HNF1α^L12H^ was achieved via site directed mutagenesis PCR, with the Flag-HNF1α^MUT^ pUltra as a template. Plasmid expansion was performed using One Shot® TOP10 competent cells, following Midiprep isolation according to manufacturer’s instructions.

#### Western Blot-IP

HEK293T cells were transfected with the cloning constructs using PEI at a 1:3 proportion. At the end of each procedure, proteins were harvested with NP-40 buffer with the addition of protease inhibitors (NaF 0.1M, PMSF 10mM, Orthovanadate 10mM, Protease inhibitor cocktail). Lysis buffer was left to act for 30 min at 4C, after which cells were centrifuged at 14000 rpm for 10 min at 4C in order to pellet cell debris. Protein supernatant was quantified using Bradford assay to ensure a consistent protein loading and either stored at −80C or directly prepared for electrophoresis. Samples were prepared by adding 4x Laemmli buffer and boiling at 99C for 5 min. For IP output, an average of 1000μg of protein was incubated O/N with 50ul anti-Flag beads (M8823, Sigma-Aldrich) at 4C, shaking. The next day, the excessive proteins/beads were washed four times using NP-40 buffer and samples were prepared for loading by boiling for 10 min at 70C. Gel electrophoresis was performed in casted 10-12% gels, separating 40-80μg of protein for 1hr 45 min at 110V. Proteins were transferred on PVDF membranes using the Turbo Transfer system at 13V for 7 min. Blocking was performed with 5% milk for an hour, after which primary antibodies were incubated O/N at 4C. The next day, the membranes were washed three times with TBS-Tween 20% (TBS-T) and incubated for 1h with the respective matching species HRP-conjugated secondary. Then, the membranes were washed again for three times with TBS-T before ECL development. ECL was left to act for 1-3 mins and signal was acquired using a Chemidoc (Bio-rad).

#### Chromatin immunoprecipitation

HEK293T were plated at day0 in 10 mm dishes and transfected the next day with the different construct combinations. 24h after transfection, each sample was crosslinked for 15 min at RT using PFA at a 1% final concentration. The reaction was then neutralized for 5 min with 125mM Glycine. Cells were then scraped and collected in falcon tubes, washed twice with 1x PBS and lysed using IP buffer (150 mM NaCl, 50 mM Tris-HCl pH 7.5, 5 mM EDTA, NP-40 0.5% vol/vol, Triton X-100 1.0% vol/vol) supplemented with protease inhibitor cocktails (Sigma Aldrich). Cell material was then washed twice using IP buffer and centrifugation at 12000g for 90 secs. Pellet was then resuspended to a final volume of 750ul IP buffer and subjected to 6 cycles of 10s sonication using Elmasonic S10 ultrasonic water bath (Camsonix). Sheared chromatin was then split for IP and mock IP, and Input DNA isolation. Mock IP was performed using A/G beads only (Thermo Scientific), while IP was performed using A/G beads loaded with anti HNF1β CHIP validated antibody (#720259, Thermo Scientific). After overnight incubation of the samples at 4C, samples were centrifuged at 12000g, beads were washed 5 times using IP buffer and resuspended to a final volume of 40ul. A 1:1 volume of 10% Chelex-100 (142-1253, Biorad) was added to all samples (IP, mock IP and DNA isolated from input samples), which were then vortexed for 10s before boiling at 98C for 10 min. Once samples cooled from the boiling, the optional step of Proteinase K digestion was performed by adding 1ul of 20 mg/ml Proteinase K and incubating for 30 min at 55C, shaking at 1000 rpm. A re-boiling step at 98C for 10 min was performed to inactivate Proteinase K. The DNA of each condition was then isolated by centrifuging at 12000g for 1.30 min at 4C, collecting the supernatant, washing the beads-chelex or chelex mix with an 100ul of ultrapure water, centrifuging again at 12000g for 1.30 min at 4C and adding the supernatant to the previously collected one. Finally, 2ul of isolated DNA was used for each RT-qPCR reaction (See RT-qPCR section).

#### Lentiviral production and infection

Lentiviral particles were produced by transfecting HEK293T with the plasmids pMD2.G, psPAX2 and the transgene-pUltra plasmids in the proportion 1:4,1:5,4 and using PEI 1:3. Supernatant containing lentivirus was collected at 48 and 72h, cleared of cell debris via centrifugation at 500g for 5 min and filtration through a 0.45μM filter. Lentiviral particles were left to concentrate using Lenti-X concentrator at 4C O/N. The next day, the virus was further concentrated via centrifugation at 1500 rpm for 45 min at 4C and then resuspended in Optimem. Viral titer was determined using HEK cells before use. To generate stable lines, cells were plated in 2D at day 0 at a density allowing a confluence of 70-80% for the next day. Cells were exposed to the virus resuspended in fresh media with the addition of 1:1000 polybrene to aid viral function. At Day 2, viral media was removed, and cells were monitored until GFP was expressed. Upon fluorophore expression, cells were FACS-sorted to isolate transgene expressing cells and purity was assessed.

#### Exome-sequencing

Exome sequencing files were provided by HipSci through the European Genome-Phenome Archive (EGA) interface. Raw files were filtered for higher confidence variants using Impute2 (Howie et al., 2009) by HipSci. Non-synonymous single-nucleotide variants (nsSNVs) from the imputed and phased files were mapped to gene and protein sequences using the ANNOtate VARiation (ANNOVAR) tool (Wang et al., 2010) to identify and confirm the p291fsinsC mutation in the MODY3 patient iPSC lines.

#### RNA-sequencing

Poly-A+ RNA was isolated from total RNA and cDNA libraries were constructed according to standardized Illumina protocols performed by Genewiz. High-throughput sequencing was performed using an Illumina HiSeq 2000 sequencer. At least 30 million 150 bp paired reads were obtained per sample with a mean quality score of 35-37. The raw RNA-sequencing read files were processed using the research computing facility at King’s College London, Rosalind (https://rosalind.kcl.ac.uk). RNA-sequencing reads were trimmed from adapters using Trimmomatic/0.39 (Bolger et al., 2014) and quality control was done using FASTQC/0.11.8 (Andrews, 2010). Reads were mapped to a concatenated genome sequence of human GRCh38/hg38 using STAR/2.4.2 (Dobin and Gingeras, 2015). Transcript abundance was measured using featureCount in Subread package (Liao et al., 2013). The DESeq2 package (Love et al., 2014) was used to identify differentially expressed genes. Volcano plots were created using the EnhancedVolcano package, heatmaps were made using the heatmap.2 function in the gplots package and PCA was performed using the plotPCA function in the DESeq2 package in R3.5.5.

### Quantification and statistical analysis

All differentiation experiments represent at least three biological replicates with similar results. All the graphs show mean values per group, with error bars representative of S.E.M. Specific P-values for each figure are represented in the graphs. Two-tailed Student’s t-test was performed for Figures 1, 2, and 4E and one-way ANOVA followed by Tukey’s test was performed for Figures 3, 4C, 5, 6, 7, S6, and S7, two-way ANOVA followed by Sidak’s multiple comparison test for Figure 4F. Statistical analysis was performed using the software GraphPad Prism 8. Differentially expressed genes were generated using the negative binomial generalized linear models for expressed genes in DESeq2 and Wald test to compare 2 groups in Figures 5 and S6. Sample sizes are reported in each figure legend. Image quantification was done through manual counting, using Imaris 9.2.0 or ImageJ 2.1.0.

## Data Availability

• All data are available from the lead contact at request. All high-throughput sequencing data, both raw and processed files, have been deposited in NCBI’s Gene Expression Omnibus and are accessible under accession number: GEO: GSE166822. • All original code has been deposited at Zenodo and is publicly available as of the date of publication (Zenodo: https://doi.org/10.5281/zenodo.5834882). • Any additional information required to reanalyze the data reported in this paper is available from the lead contact upon request.

## Supplemental information

Document S2. Tables S1–S3

Document S3. Article plus supplemental information
