# Supplementary material for: An HNF1α truncation associated with maturity-onset diabetes of the young impairs pancreatic progenitor differentiation by antagonizing HNF1β function
Source: Cell Rep. 2022 Mar 1;38(9):110425. doi: 10.1016/j.celrep.2022.110425 (PMC8905088; doi:10.1016/j.celrep.2022.110425)
Supplement: Document S1. Figures S1–S7 [file mmc1.pdf]

Cell Reports, Volume *38*

## Supplemental information

# An HNF1α truncation associated with maturity-onset diabetes of the young impairs pancreatic progenitor differentiation by antagonizing HNF1β function

**Ana-Maria Cujba, Mario E. Alvarez-Fallas, Sergio Pedraza-Arevalo, Anna Laddach, Maggie H. Shepherd, Andrew T. Hattersley, Fiona M. Watt, and Rocio Sancho**

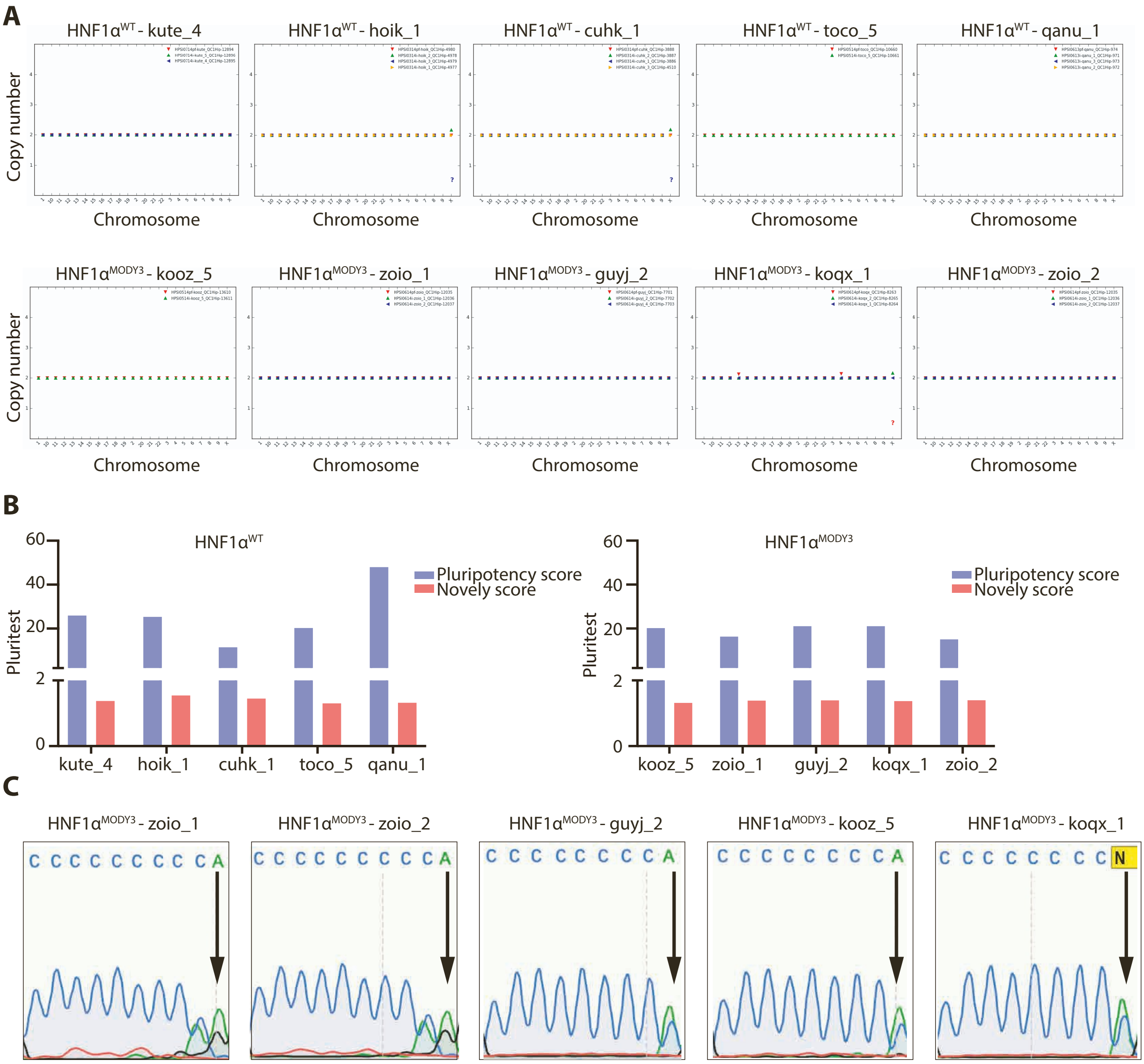


D

| IPSC line | Chr | Start | End | Ref | Alt | Func.refGene | Gene.refGene | ExonicFunc.refGene | AAChange.refGene |
|---|---|---|---|---|---|---|---|---|---|
| ZOIO2 | 12 | 121432117 | 121432117 | - | C | exonic | HNF1A | frameshift insertion | HNF1A:NM_000545:exon4:c.865dupC:p.G292Rfs*23,HNF1A:NM_001306179:exon4:c.865dupC:p.G292Rfs*23 |
| ZOIO1 | 12 | 121432117 | 121432117 | - | C | exonic | HNF1A | frameshift insertion | HNF1A:NM_000545:exon4:c.865dupC:p.G292Rfs*23,HNF1A:NM_001306179:exon4:c.865dupC:p.G292Rfs*23 |
| KOOZ5 | 12 | 121432117 | 121432117 | - | C | exonic | HNF1A | frameshift insertion | HNF1A:NM_000545:exon4:c.865dupC:p.G292Rfs*23,HNF1A:NM_001306179:exon4:c.865dupC:p.G292Rfs*23 |
| GUYJ2 | 12 | 121432125 | 121432125 | - | C | exonic | HNF1A | frameshift insertion | HNF1A:NM_000545:exon4:c.872dupC:p.G292Rfs*23,HNF1A:NM_001306179:exon4:c.872dupC:p.G292Rfs*23 |
| KOQX1 | 12 | 121432125 | 121432125 | - | C | exonic | HNF1A | frameshift insertion | HNF1A:NM_000545:exon4:c.872dupC:p.G292Rfs*23,HNF1A:NM_001306179:exon4:c.872dupC:p.G292Rfs*23 |

**Figure S1. Quality control of human iPSC lines used in this study. Related to Figure 1.**

A) Karyotype data indicating normal copy numbers for all chromosomes in the HNF1α$^{WT}$ and HNF1α$^{MODY3}$ iPSC lines used in this study, derived from the HipSci database. B) Pluritests indicating high pluripotency scores and low novelty scores across lines for the five HNF1α$^{WT}$ and HNF1α$^{MODY3}$ iPSC lines used in this study from the HipSci database. C) Sequencing analysis indicating the heterozygous p291fsinsC mutation occurring in all the HNF1α$^{MODY3}$ patient iPSC lines used in this study by arrows. D) Exome sequencing analysis of HNF1α$^{MODY3}$ patient iPSC lines used in this study, depicting the occurrence of the p291fsinsC frameshift mutation that results in an amino acid change from glycine (G) to arginine (R) at codon 291 in exon4.

**A**

| iPSC line | Sex | Age at iPS derivation | Age at diagnosis | Clinical features |
|---|---|---|---|---|
| ZOIO1 ZOIO2 | F | 75-79 | 43 | BMI 25, Hba1c 58mmol/mol, HbA1c = 7.1%, retinopathy, hypercholesterolaemia, hypertension, cholesterol 4.8 mmol/L; Treatment: Gliclazide, Sitagliptin |
| GUYJ2 | F | 50-54 | 43 | BMI 24; Treatment: diet only |
| KOQX1 | F | 25-29 | 18 | BMI 24, HbA1c 45mmol/mol, cholesterol 4.2 mmol/L; Treatment: Glibenclamide |
| KOOZ5 | F | 40-44 | 30 (during pregancy) | BMI 27, HbA1c 98mmol/mol, HbA1c = 7.1%, hypertension, cholesterol 7.3 mmol/L: Treatment:Gliclazide, Humulin |

Glycated hemoglobin (Hba1c) levels: Normal Hba1c: 4% - 5.6%; < 42mmol/l; High chance of getting diabetes: 5.7% - 6.4%; 42-47mmol/l; Diabetes > 6.5%; >48mmol/l (clinician info)
Non-HDL (bad cholesterol): healthy level 4 mmol/L or bellow (NHS website)
BMI: between 18.5 and 24.9 - healthy weight range; between 25 and 29.9 - overweight range (NHS)

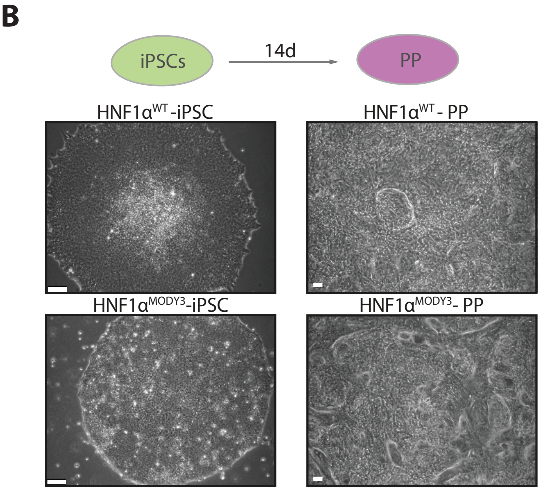


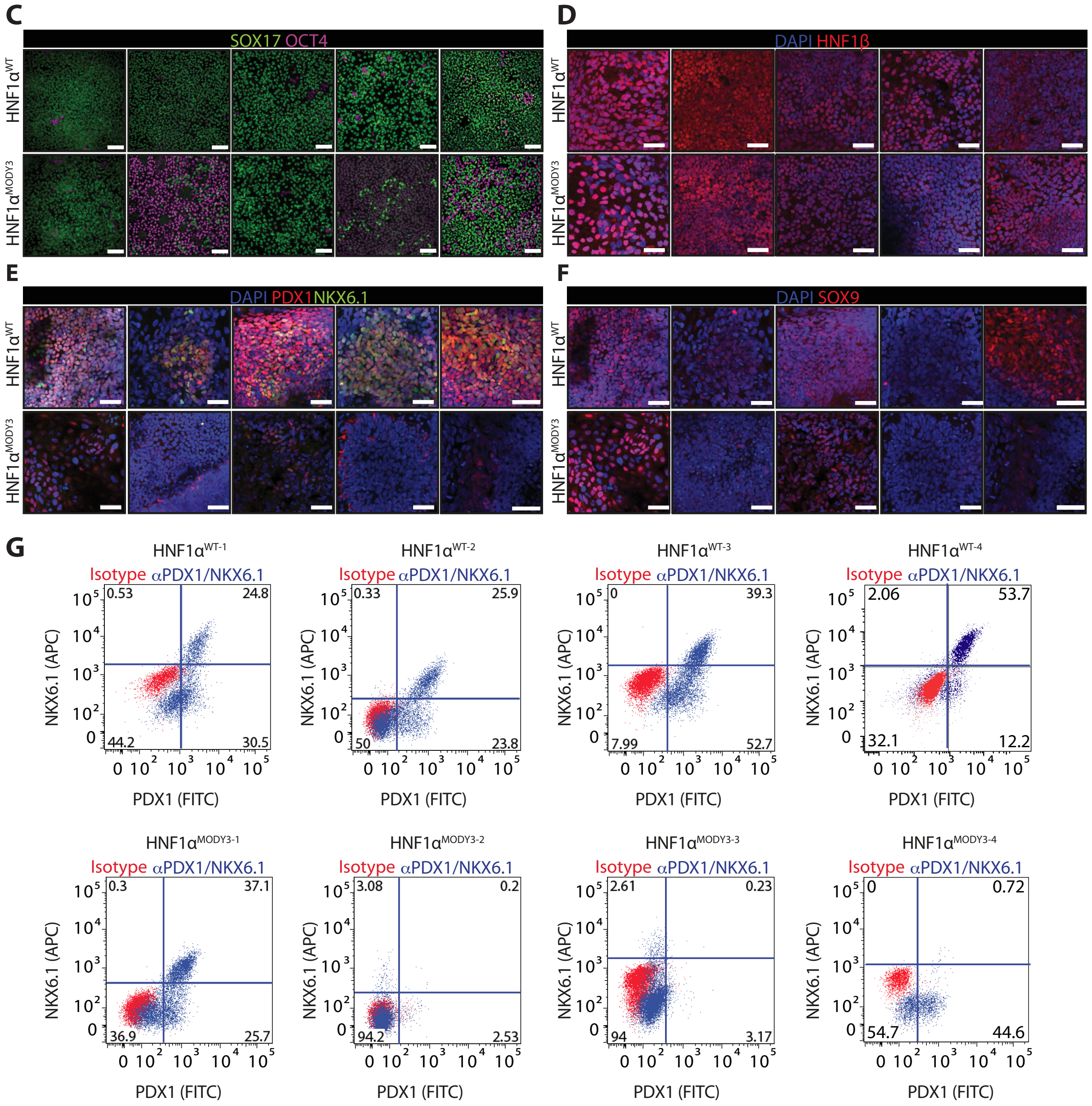


**Figure S2. Differentiation of patient HNF1α$^{MODY3}$ iPSC lines into pancreatic progenitors is impaired compared to healthy donor HNF1α$^{WT}$ iPSC lines. Related to Figure 1.**

A) Clinical features of the patients from which the HNF1α$^{MODY3}$ iPSC lines used in this study were derived. B) Schematic of the differentiation of HNF1α$^{WT}$/HNF1α$^{MODY3}$ iPSCs towards PPs and respective brightfield images for those stages. C) IF analysis of OCT4 and SOX17 at DE stage for all HNF1α$^{WT}$ and HNF1α$^{MODY3}$ differentiated lines used for quantification in Figure 1E. D) IF analysis of HNF1b at PF stage for all HNF1α$^{WT}$ and HNF1α$^{MODY3}$differentiated lines used for quantification in Figure 1E. E) IF analysis of PDX1, NKX6.1 at the PP stage for all HNF1α$^{WT}$ and HNF1α$^{MODY3}$differentiated lines used for quantification in Figure 1H. F) IF analysis of SOX9 at the PP stage for all HNF1α$^{WT}$ and HNF1α$^{MODY3}$differentiated lines used for quantification in Figure 1H. G) Representative flow cytometry analysis of PPs obtained from the other cell lines used for quantification in Fig. 1J. Scale bars, 100μm (B), 50μm (C, D, E, F).

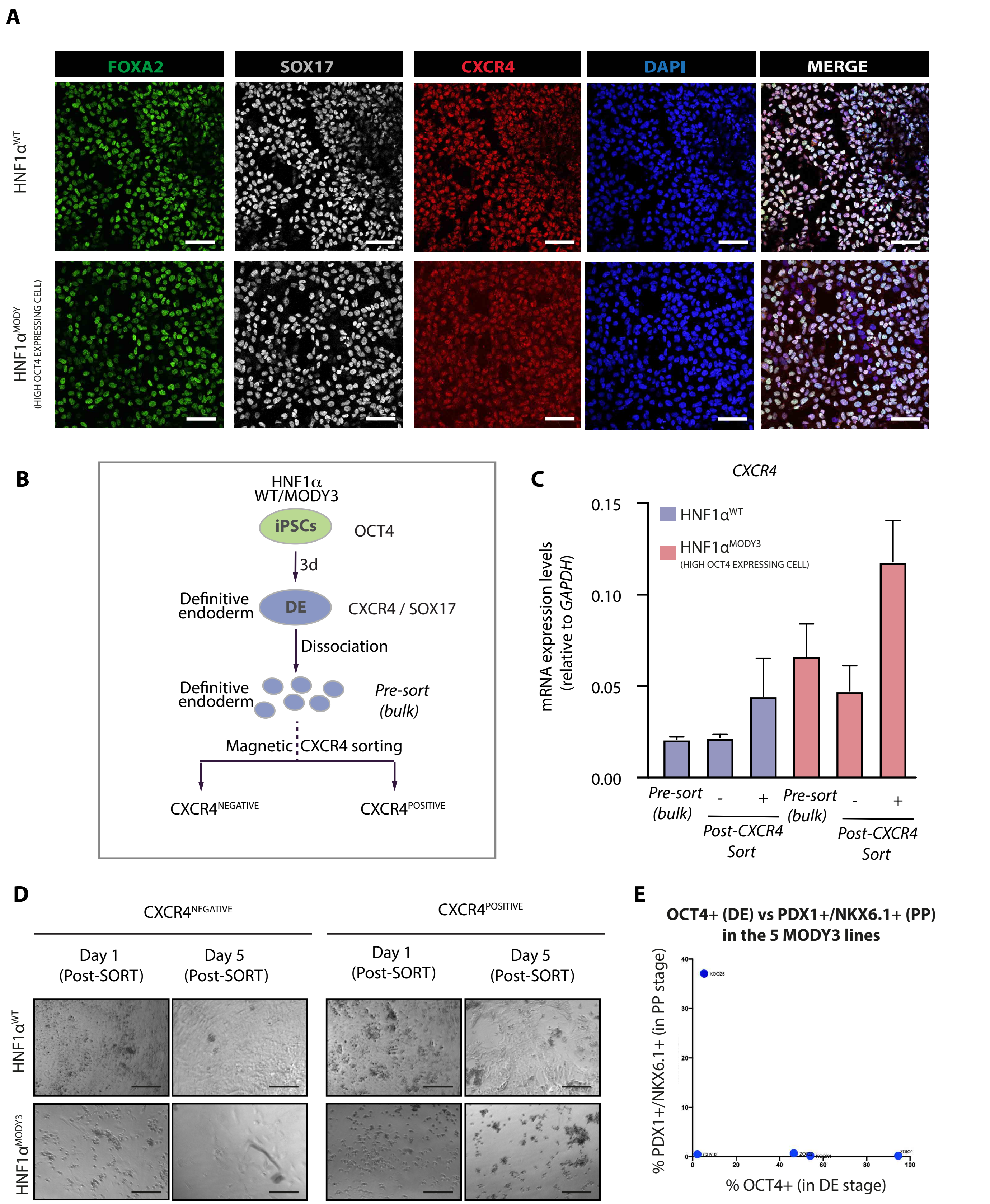


**Figure S3. PP differentiation after CXCR4 sorting at DE stage in HNF1α$^{WT}$ and HNF1α$^{MODY}$ lines . Related to Figure 1.**

A) Immunofluorescense for SOX17 and CXCR4 in HNF1α$^{WT}$ and HNF1α$^{MODY3}$ iPSCs lines after differentiation to DE. B) Schematic diagraman of the CXCR4 sorting strategy. C) qPCR analysis of *CXCR4* in different fractions from the CXCR4 sorting. D) Bright field images of CXCR4$^{NEGATIVE}$ and CXCR4$^{POSTIVE}$ fractions 1 day and 5 days after plating. E) Scatter plot representing the % of OCT4+ cells in DE versus the % of PDX1+/NKX6.1+ in PP stage for each individual HNF1α$^{MODY3}$ line. Scale bars, 50μm (A), 1mm (D).

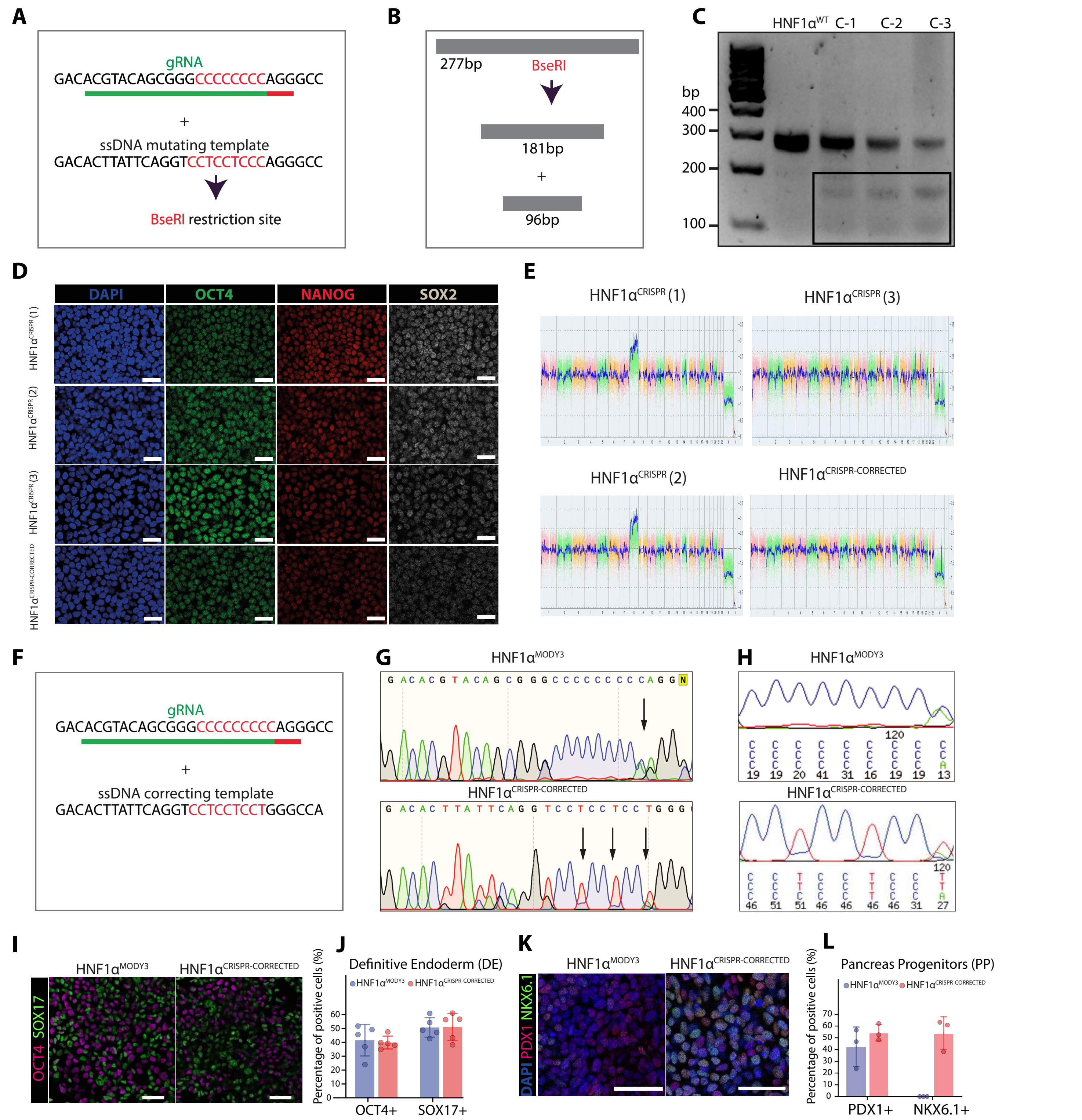


**Figure S4. CRISPR-Cas9 strategy. Related to Figure 2.**

A) Schematic depicting the gRNA that cuts adjacent to the polyC tract (PAM sequence in red) and the silently encoded ssDNA template harbouring the heterozygous p291fsinsC mutation and a new BseRI restriction site. B) Screening strategy by BseRI digest. C) Confirmation of three positive HNF1α$^{CRISPR}$ iPSC clones through BseRI digestion. D) Confirmation of pluripotency of the three positive HNF1α$^{CRISPR}$ iPSC clones and one HNF1α$^{CRISPR\text{-}CORRECTED}$ iPSC clone. E) Low pass sequencing of the CRISPR clones. F) Schematic depicting the gRNA design that cuts adjacent to the p291fsinsC mutation (PAM sequence in red) and the silently encoded ssDNA wild-type template. G) Chromatograms of the mutant HNF1α$^{MODY3}$iPSC line (top) and wild-type HNF1α$^{CRISPR\text{-}CORRECTED}$ (bottom). H) Sequencing analysis of the HNF1α$^{MODY3}$ line (top) and the HNF1α$^{CRISPR\text{-}CORRECTED}$ line (bottom) obtained through the Mixed Sequence Reader tool (http://msr.cs.nthu.edu.tw/) showing heterozygosity. I) Representative IF images of the HNF1α$^{MODY3}$ iPSC line and the HNF1α$^{CRISPR\text{-}CORRECTED}$ iPSC line after differentiation to DE stage. J) Quantification analysis afterdifferentiation to DE stage as shown in IF in I. N=5. K) Representative IF images of the HNF1α$^{MODY3}$ iPSC line and the HNF1α$^{CRISPR\text{-}CORRECTED}$ iPSC line after differentiation to PP stage. L) Quantification analysis after differentiation to PP stage as shown in IF in K. N=3. Scale bars, 30µm (D), 50µm (I, K).

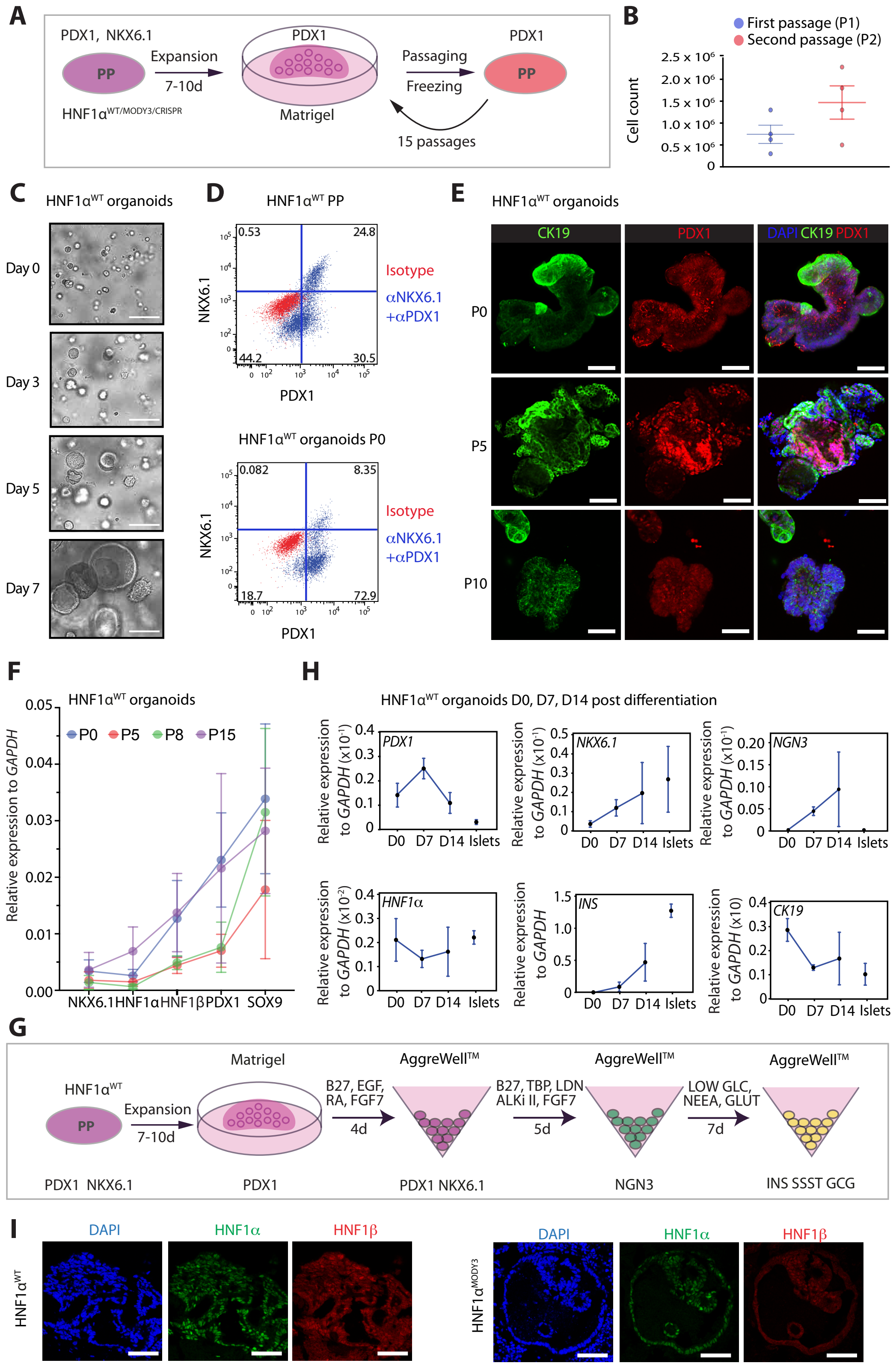


**Figure S5. Organoids derived from HNF1α$^{WT}$ pancreatic progenitors can be expanded and differentiated in-vitro in 3D. Related to Figure 3.**

A) Diagram depicting the strategy to expand progenitor organoids up to 15 passages, while undergoing multiple freeze-thaw cycles. B) Cell counts during expansion of HNF1α$^{WT}$ organoids over one passage. C) Brightfield images of expanding HNF1α$^{WT}$ organoids during a 7-day period. D) Flow cytometry analysis of PDX1+ NKX6.1+ cells derived from HNF1α$^{WT}$ progenitors at the PP stage and at the pancreatic organoid (PO) stage. E) IF analysis of PDX1 and CK19 in HNF1α$^{WT}$ organoids at passages P0, P5 and P10. F) RT-qPCR analysis of progenitor markers in HNF1α$^{WT}$ organoids at passages P0, P5 and P10. N = 3 independent experiments. G) Schematic depicting the strategy to differentiate progenitor organoids towards beta-cells in suspension. H) RT-qPCR analysis of progenitor markers PDX1, NKX6.1, NGN3, HNF1a, beta-cell marker insulin and ductal cell marker CK19 in expandable organoids in Matrigel, day 7, day 14 of the differentiation protocol and human islets. N = 3 independent experiments. I) IF analysis of HNF1α and HNF1β expression in HNF1α$^{WT}$ and HNF1α$^{MODY3}$ progenitor organoids. Scale bars, 100μm (C), 50μm (E, I). Error bars are SEM.

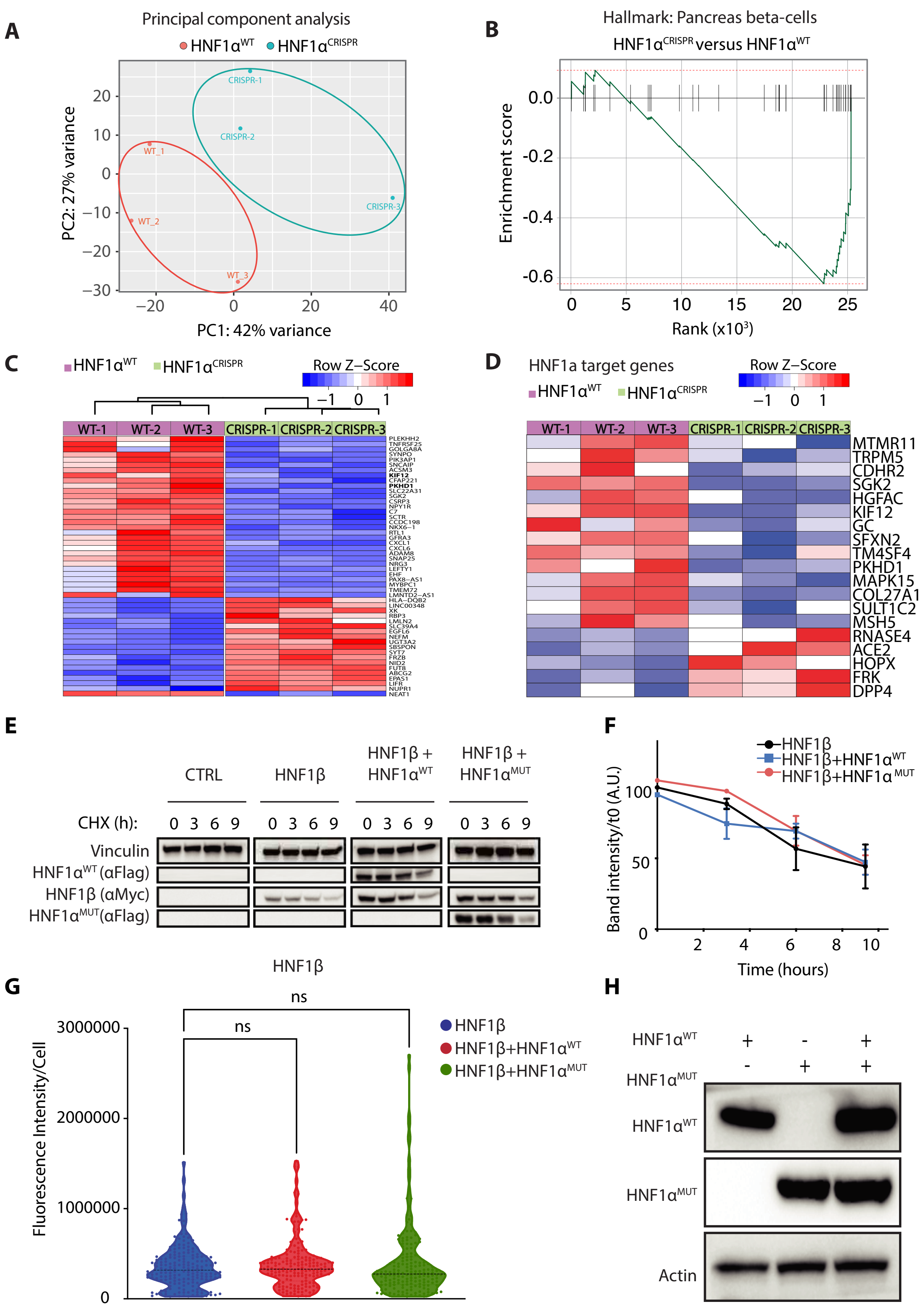


**Figure S6. Truncating HNF1α p291fsinsC mutant protein interacts with HNF1β to impair progenitor development. Related to Figure 5.**

A) Principal component analysis of the sequenced HNF1α$^{WT}$/HNF1α$^{CRISPR}$ PPs. B) Significant decrease of the enrichment score for beta-cell signature in the comparison of HNF1α$^{CRISPR}$ versus HNF1α$^{WT}$ PPs. C) Heatmap of top 50 significantly dysregulated genes in the comparison of HNF1α$^{CRISPR}$ versus HNF1α$^{WT}$ PPs. D) Heatmap of significantly dysregulated HNF1α$^{WT}$ target genes in the comparison of HNF1α$^{CRISPR}$ versus HNF1α$^{WT}$ PPs. E) Cycloheximide chase performed on HEK293T transfected with the HNF1α$^{WT}$/HNF1α$^{MUT}$ and HNF1β proteins. F) Quantification of HNF1β protein degradation kinetics, normalized on the t0 amount of protein. G) Quantification of HNF1β fluorescence intensity/cell from IF analysis in Figure 5E. H) Immunoblot of HNF1α$^{WT}$/HNF1α$^{MUT}$ proteins after independent overexpression or coexpression in HEK293T.

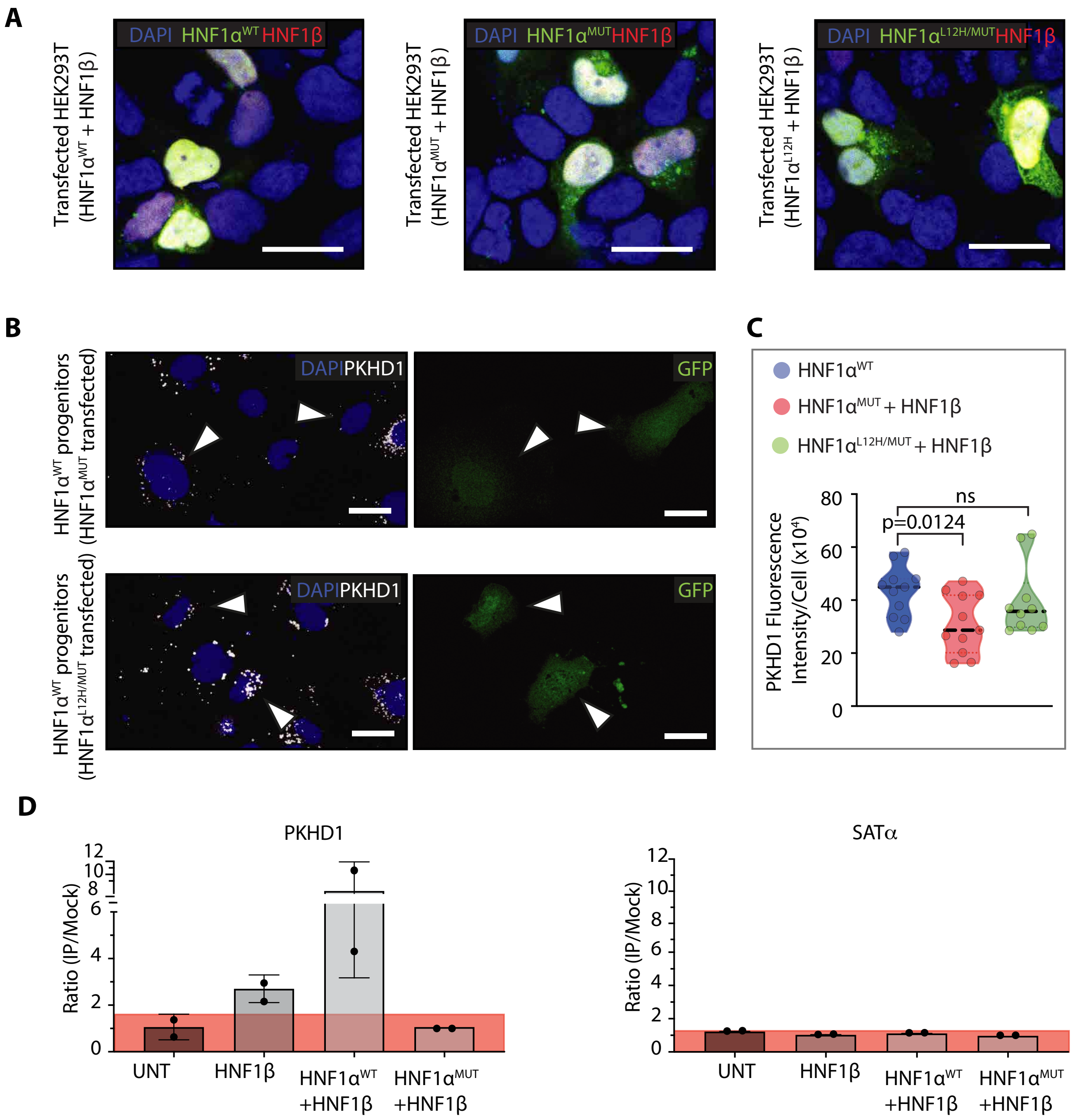


**Figure S7. Truncating HNF1α p291fsinsC mutant protein interacts with HNF1β to impair progenitor development. Related to Figure 5.**

A) IF showing the localization of transfected $HNF1\alpha^{WT}$/ $HNF1\alpha^{MUT}$/$HNF1\alpha^{L12H/MUT}$ and HNF1β proteins in HEK293T. B) IF analysis for PKHD1 performed on $HNF1\alpha^{WT}$ organoids transfected with $HNF1\alpha^{WT}$/ $HNF1\alpha^{MUT}$/$HNF1\alpha^{L12H/MUT}$ proteins. Arrowheads = transfected cells. C) Quantification of fluorescence intensity of PKHD1+ cells from IF analysis. N = 3 independent experiments. D) Chromatin immunoprecipitation assay on PKHD1 and negative control SATα. Scale bars, 100 µm (A, B), p-values shown for significant differences determined by one-way ANOVA followed by Tukey's multiple comparison test. ns = non-significant.
