# Supplementary material for: An HNF1α truncation associated with maturity-onset diabetes of the young impairs pancreatic progenitor differentiation by antagonizing HNF1β function
Source: Cell Rep. 2022 Mar 1;38(9):110425. doi: 10.1016/j.celrep.2022.110425 (PMC8905088; doi:10.1016/j.celrep.2022.110425)
Supplement: Document S2. Tables S1–S3 [file mmc2.pdf]

Supplementary Table 1. Name and sequences of the primers used in this study.

Oligo name	Oligo sequence (5' to 3')	Oligo name	Oligo sequence (5' to 3')
hOCT4_F	GTGTTCAGCCAAAAGACCATCT	hPKHD1_F	TGGCTGAATCCTGATGTGGT
hOCT4_R	GGCCTGCATGAGGGTTTCT	hPKHD1_R	TTGCAGGGATTGGCTGACTA
hFOXA2_F	GGGAGCGGTGAAGATGGA	hKIF12_F	CAAACCTGGCCCAGAGACT
hFOXA2_R	TCATGTTGCTCACGGAGGAGTA	hKIF12_R	CTCACTGCAGGGTGGCT
hSOX17_F	ACGCCGAGTTGAGCAAGA	hTWIST1_F	TCGGACAAGCTGAGCAAGAT
hSOX17_R	TCTGCCTCCTCCACGAAG	hTWIST1_R	CTCCATCCTCCAGACCGAG
hHNF1A_L	GAGGCAGAAGAACCCTAGCA	hWNT5A_F	CCTTCGCCCAGGTTGTAATT
hHNF1A_R	GACACCCCTCTCTGGATGC	hWNT5A_R	TGGTCCTGATACAAGTGGCA
hHNF1B_F	TCACAGATACCAGCAGCATCAGT	hJAG1_F	CTTGCAAACTCCCAGGTGAC
hHNF1B_R	GGGCATCACCAGGCTTGTA	hJAG1_R	TTGAGACACGGCTGATGAGT
hSOX9_F	GAACGCACATCAAGACGGAG	hE-CAD_F	CACCACGGGCTTGGATTTTG
hSOX9_R	AGTTCTGGTGGTCGGTGTAG	hE-CAD_R	TGGGGGCTTCATTCACATCC
hNKX6-1_F	CACACGAGACCCACTTTTTC	hGCG_F	TTCCCAGAAGAGGTCGCCATTGTT
hNKX6-1_R	CCGCCAAGTATTTTGTTTGT	hGCG_R	CAACCAGTTTATAAAGTCCCTGGCGG
hPDX1_F	CACATCCCTGCCCTCCTAC	hSST_F	GAGAATGATGCCCTGGAACCTGAAGA
hPDX1_R	GAAGAGCCGGCTTCTCTAAAC	hSST_R	ATTCTTGCAGCCAGCTTTGCGT
hNGN3_F	CTCGGACCCCATTCTCTCTT	hINS_R	CACAATGCCACGCTTCTG
hNGN3_R	CTTCTGGTCGCCAAGTTCA	hINS_F	AGGCTTCTTCTACACACCCAAG
hCK19_F	GCCACTACTACACGACCATGG	hGAPDH_F	TTGCTTGTAGCCAAATTCGTTG
hCK19_R	CAAACTTGGTTCGGAAGTCAT	hGAPDH_R	ATTGCCCTCAACGACCACTTT

Supplementary Table 2. CRISPR-Cas9 strategy for correcting and inducing p291fsinsC mutation in human iPSC lines.

gRNAs and ssDNA template to induce p291fsinsC mutation in $HNF1\alpha^{WT}$ line kute4	gRNA-WT(upper): 5'-**CACCg**acgtacagcgggcccccccc-3'
	gRNA-WT(lower): 5'-**AAAC**ggggggggcccgctgtacgt**c**-3'
	ssDNA template (for inducing p291finsC mutation): 5'CGGCGCAAAGAAGAAGCCTTCCGGCACAAGCTGGCCATGGAC**ACTTATTCAGGTCCTCCTCCC**AGGGCCAGGCCCGGGACCTGCGCTGCCCGCTCACAGCTCCCCTGGCCTGCCTCCACCT-3'
gRNAs and ssDNA template to correct p291fsinsC mutation in $HNF1\alpha^{MODY3}$ line zoio2	gRNA-MUT (upper): 5'**CACCg**cgtacagcgggccccccccc-3'
	gRNA-MUT (lower): 5'**AAAC**gggggggggcccgctgtacgc-3'
	ssDNA template (for correcting p291finsC mutation): 5'CGGCGCAAAGAAGAAGCCTTCCGGCACAAGCTGGCCATGGACACTTATTCAGGT**CCTCCTCCT**GGGCCAGGCCCGGGACCTGCGCTGCCCGCTCACAGCTCCCCTGGCCTGCCTCCACCT-3'

Supplementary Table 3. Oligonucleotides used for construct cloning and point mutagenesis

BamHI-Flag-F: 5’- TGCTTAGGATCC ATGGATTACAAGGATGACGACGATAAG
Flag-HNF1α-F: 5’ - CAAGGATGACGACGATAAGGTTTCTAAACTGAGCCAGCT
EcoRI-HNF1α-R: 5’- TGCTTAGAATTCTTACTGGGAGGAAGAGGCCATCTGGGT
EcoRI-HNF1α-MUT-R 5’ - TGCTTAGAATTCCCTGGCCCTGGGGGGGGGCCCGCTGTA
BamHI-Myc-F: 5’- TGCTTAGGATCCATGGAACAAAAACTCATCTCAGAAGAGGATCTG
Myc-HNF1α-F: 5’- CTCATCTCAGAAGAGGATCTGGTGTCCAAGCTCACGTCGCT
EcoRI-HNF1α-R 5’ - TGCTTAGAATTCTCACCAGGCTTGTAGAGGACACTGT
L12H mutation: Forward 5’ - CTGCAGACGGAGCACCTGGCGGCCC - 3’,<br>Reverse 5’ - GGGCCGCCAGGTGCTCCGTCTGCAG - 3’
